# Quantifying the Stability of the Hydronium Ion in Organic Solvents With Molecular Dynamics Simulations

**DOI:** 10.3389/fchem.2019.00439

**Published:** 2019-06-19

**Authors:** Alex K. Chew, Reid C. Van Lehn

**Affiliations:** Department of Chemical and Biological Engineering, University of Wisconsin – Madison, Madison, WI, United States

**Keywords:** acid-catalyzed reactions, solvent effects, biomass conversion, hydronium ion, classical molecular dynamics simulation, solvation free energy

## Abstract

The solution-phase stability of the hydronium ion catalyst significantly affects the rates of acid-catalyzed reactions, which are ubiquitously utilized to convert biomass to valuable chemicals. In this work, classical molecular dynamics simulations were performed to quantify the stability of hydronium and chloride ions by measuring their solvation free energies in water, 1,4-dioxane (DIOX), tetrahydrofuran (THF), γ-valerolactone (GVL), N-methyl-2-pyrrolidone (NMP), acetone (ACE), and dimethyl sulfoxide (DMSO). By measuring the free energy for transferring a hydronium ion from pure water to pure organic solvent, we found that the hydronium ion is destabilized in DIOX, THF, and GVL and stabilized in NMP, ACE, and DMSO relative to water. The distinction between these organic solvents can be used to predict the preference of the hydronium ion for specific regions in aqueous mixtures of organic solvents. We then incorporated the stability of the hydronium ion into a correlative model for the acid-catalyzed conversion of 1,2-propanediol to propanal. The revised model is able to predict experimental reaction rates across solvent systems with different organic solvents. These results demonstrate the ability of classical molecular dynamics simulations to screen solvent systems for improved acid-catalyzed reaction performance.

## Introduction

**Figure 1:**
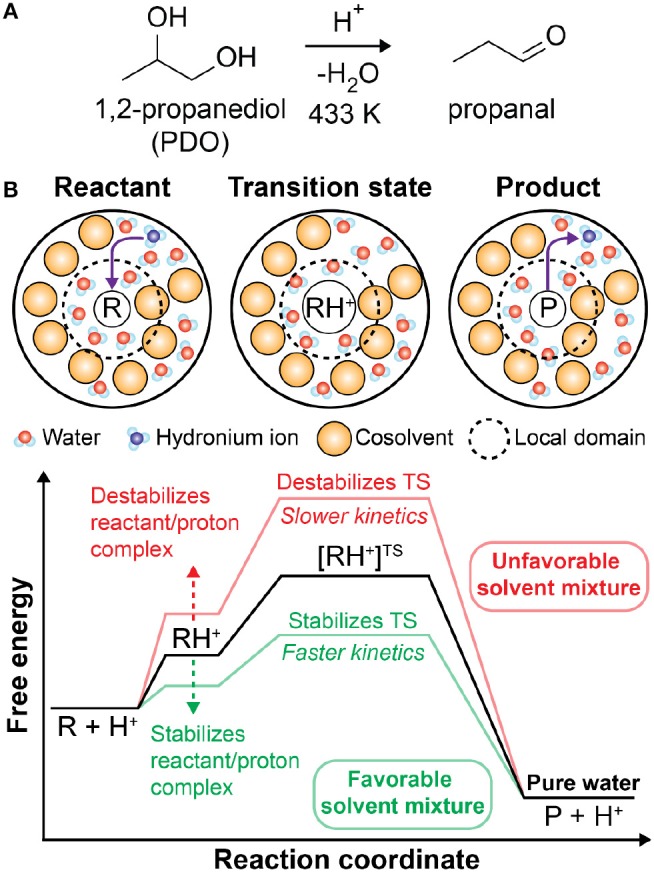
**(A)** Brønsted acid-catalyzed reaction of 1,2-propanediol (PDO) to propanal. **(B)** Schematic of acid-catalyzed reactions in mixed-solvent environments. The reaction proceeds through a charged transition state (TS) formed from the protonation of the reactant by a hydronium ion catalyst. The corresponding free energy diagram schematically depicts the influence of mixed-solvent environments (red and green lines) on acid-catalyzed reactions relative to pure water (black line). This free energy landscape is representative of 1,2-propanediol dehydration based on prior computational findings (Mellmer et al., 2018). For different acid-catalyzed reactions, the relative free energies of the various states may differ (Mellmer et al., 2018), but general features should be similar.

The catalytic upgrading of biomass (e.g., wood, crops, etc.) is a promising strategy to obtain valuable chemicals from renewable resources while limiting waste products (Huber et al., 2006; Stöcker, 2008; Tock et al., 2010; Shuai and Luterbacher, 2016; Nguyen et al., 2017; Walker et al., 2019). For example, cellulose, one of the primary components of lignocellulosic biomass, can be converted through a series of dehydration and hydrolysis reactions to form 5-hydroxymethylfurfural, a platform chemical for fuels and other commodity chemicals (Chheda et al., 2007; Corma et al., 2007; Román-Leshkov et al., 2007; Pagan-Torres et al., 2012; Mellmer et al., 2015; He et al., 2017). These reactions are typically performed in aqueous solution where extensive control over reaction kinetics and selectivity is available by tuning the temperature, catalyst, and solvent composition (Huber et al., 2006; Chheda et al., 2007; Román-Leshkov et al., 2007; Mellmer et al., 2014b; Motagamwala et al., 2016; He et al., 2017; Won et al., 2017; Sener et al., 2018). Solution-phase biomass conversion reactions ubiquitously require an acidic proton catalyst (H^+^), which exists in solution as a hydronium ion (H_3_O^+^). In homogenous reactions, the catalyst is obtained from the addition of a Brønsted acid (He et al., 2017) and the reactions follow a specific catalysis mechanism since a protonated solvent is the catalyst. Figure 1A shows an example reaction for the acid-catalyzed dehydration of 1,2-propanediol to propanal, which is representative of acid-catalyzed reactions for biomass-derived model compounds (Mellmer et al., 2018; Walker et al., 2019). In these reactions, the hydronium ion catalyst (H_3_O^+^) protonates the reactant (R) to form a reactant/proton complex (RH^+^). The reaction proceeds to a charged transition state ([RH^+^]^TS^) and subsequently forms the product (P) with the hydronium ion reformed (Figure 1B). The relative stabilities of the reactant, transition state, and catalyst in solution are thus critical for determining reaction kinetics (Shuai and Luterbacher, 2016). Understanding how these solvent effects influence reaction kinetics is necessary to guide the optimization of solvent compositions and reactor conditions and maximize the productivity of biomass conversion reactions.

Previous studies have found that mixtures of water and organic, polar aprotic cosolvents (i.e., mixed-solvent environments) can increase or decrease the rates of Brønsted acid-catalyzed reactions depending on the stability of the acid catalyst (Mellmer et al., 2014b, 2018; Shuai and Luterbacher, 2016; Sener et al., 2018). One mechanism by which the solvent composition affects catalyst stability is by shifting the acid dissociation equilibrium, which is quantified by the acid disassociation constant (K_a_). For example, weak acids with small K_a_ values, such as formic acid or acetic acid, were found to decrease acid-catalyzed reaction rates in mixed-solvent environments compared to pure water due to the reduced availability of catalytic hydronium ions (Mellmer et al., 2014b). Conversely, strong acids with large K_a_ values, such as triflic acid, dissociate in a small fraction of water and were found to improve xylose conversion reaction rates by 40-fold in 90 wt% γ-valerolactone (Mellmer et al., 2014b), suggesting an alternative role for the solvent. Based on combined classical and *ab initio* molecular dynamics (MD) simulations, Mellmer et al. found that the hydronium ion catalyst in mixed-solvent systems is destabilized in the bulk solvent relative to the local solvent domain of the reactant due to unfavorable interactions between the hydronium ion and the cosolvent (Mellmer et al., 2018). As a result, the acid catalyst is thermodynamically driven to water-enriched local solvent domains formed by hydrophilic reactants when a high mass fraction of the organic phase is present, effectively lowering activation energy barriers and increasing reaction rates relative to pure water (Figure 1B). Together, these results indicate that the solvent composition can modulate reaction kinetics by both modulating catalyst availability and the interactions of the catalyst with the reactant.

Building upon these studies, we hypothesized that acid-catalyzed reaction rates correlate with the formation of water-enriched local solvent domains because the catalyst is assumed to be stabilized by interactions with water and thus the formation of water-enriched local solvent domains would drive the partitioning of the catalyst to the reactant (Mellmer et al., 2018; Walker et al., 2019). We derived a correlative model that used descriptors derived from classical MD simulations to predict experimental reaction kinetics for seven biomass-derived model compounds in aqueous mixtures of dioxane and γ-valerolactone (Walker et al., 2019). While reaction free energies are typically determined from *ab initio* level studies (Caratzoulas and Vlachos, 2011; Mellmer et al., 2018), this hypothesis allowed us to use classical MD simulations to more rapidly screen through multiple solvent compositions and reactions. However, the assumption that the hydronium ion preferentially interacts with water is not always true. For instance, more basic organic solvents, such as dimethyl sulfoxide (DMSO), have been shown to participate in the reaction mechanism by stabilizing the proton (Mellmer et al., 2018). The addition of DMSO has also been shown to diminish acid-catalyzed conversion of *tert*-butanol (Mellmer et al., 2018), indicating that addition of organic solvents can also destabilize the reactant/proton complex, raise energy barriers, and consequently slow reaction kinetics relative to pure water (Figure 1B). Therefore, understanding and quantifying the thermodynamic stability of the hydronium ion in mixed-solvent environments is essential to accurate predictions of acid-catalyzed reaction kinetics.

It is experimentally difficult to directly measure the free energy of an isolated hydronium ion in solution since electroneutrality must be maintained (Reif and Hünenberger, 2011). To obtain single-ion thermodynamics, non-classical techniques such as atomic and molecular spectroscopy combined with statistical mechanics are utilized (Hunenberger and Reif, 2011). To broaden the range of possible systems, computational tools have been developed to model the hydronium ion and isolate the role of the solvent on the acid catalyst (Varghese and Mushrif, 2019). For solution-phase reactions, we quantify the stability of the acid catalyst in terms of its solvation free energy, or the free energy for introducing the catalyst in solution. The solvation free energy accounts for interactions between the catalyst and solvent (e.g., hydrogen bonding, ion-dipole interactions, and van der Waals forces) and the solvent reorganization necessary to accommodate the catalyst. Solvation free energies are also important in determining the partitioning of ions between different phases (Duignan et al., 2017). Typically, *ab initio* level simulations are performed to accurately compute solvation free energies of the acid catalyst (Tunon et al., 1993; Tawa et al., 1998; Mejias and Lago, 2000; Pliego and Riveros, 2001; Kelly et al., 2007). However, these simulations are computationally expensive and thus challenging to perform for multiple solvent compositions. Recently, Bonthuis et al. developed a classical hydronium ion model that accurately reproduces experimental solvation free energies in pure water (Bonthuis et al., 2016). We thus hypothesize that this classical hydronium ion model can be used to compute solvation free energies of the acid catalyst and leverage the computational efficiency of MD simulations to screen stability in different solvent compositions, assuming that the hydronium ion maintains its structure in these solvents. These calculations can then be used to predict the relationship between solvent composition and reaction kinetics for acid-catalyzed reactions.

Herein, we use classical MD simulations to study the stability of a hydronium ion in six organic polar aprotic cosolvents: dioxane (DIOX), tetrahydrofuran (THF), γ-valerolactone (GVL), N-methyl pyrrolidine (NMP), acetone (ACE), and dimethyl sulfoxide (DMSO). We also study the stability of a chloride ion in the same solvents to calculate the effect of the conjugate base. We use previous literature values for the reaction rates of the acid-catalyzed conversion of 1,2-propandiol (PDO) as a model reaction to study the influence of the different cosolvents. Since our previous work found favorable agreement between MD simulation-derived descriptors with experimental reaction rates without mechanistic details of the reaction (Walker et al., 2019), we focus on studying how water-enrichment (or cosolvent-enrichment) can improve reaction performance by favorably facilitating a hydronium ion. We then quantify the stabilities of the hydronium and chloride ions in pure and mixed-solvent environments by computing the solvation free energies. We find that the free energy for transferring a hydronium ion from pure water to organic solvent can distinguish between solvents that favorably (NMP, ACE, DMSO) and unfavorably (DIOX, THF, GVL) solvate the acid catalyst. With this information, we improve our previously developed correlative model for the conversion of PDO (Walker et al., 2019) by including a cosolvent-specific descriptor that incorporates information about the stability of the hydronium ion in the solvent system.

## Methods

Classical MD simulations were performed using GROMACS 2016 (Páll et al., 2015). We used the classical hydronium and chloride ion models parameterized by Bonthuis et al. (2016), which have been found to reproduce experimental solvation free energies in pure water systems modeled using the Single Point Charge/Extended (SPC/E) water model (Berendsen et al., 1987). Bond constraints for the hydronium ion were modified to improve simulation performance by using the more efficient LINCS constraint algorithm (Hess et al., 1997) instead of the SHAKE constraint algorithm (Ryckaert et al., 1977) (Supplementary Section 1, Supplementary Material). PDO and all cosolvents were parameterized using the CGenFF/CHARMM36 forcefields (Vanommeslaeghe et al., 2009; Yu et al., 2012; Best et al., 2013), while water was modeled using the SPC/E model (Berendsen et al., 1987). For all simulations, Verlet lists were generated using a 1.2 nm neighbor list cutoff. Van der Waals interactions were modeled with a Lennard-Jones (LJ) potential with a 1.2 nm cutoff that was smoothly shifted to zero between 1.0 and 1.2 nm. Electrostatic interactions were calculated using the smooth Particle Mesh Ewald method with a short-range cutoff of 1.2 nm, grid spacing of 0.12 nm, and 4th order interpolation. Bonds were constrained using the LINCS algorithm. All thermostats used a 1.0 ps time constant and all barostats used a 5.0 ps time constant with an isothermal compressibility of 5.0 ×10^−5^ bar^−1^.

**Figure 2:**
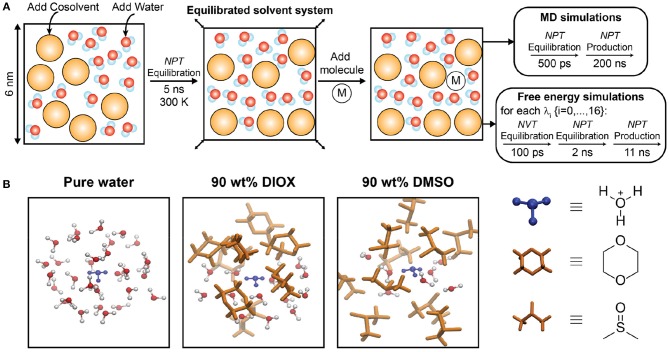
**(A)** Schematic representation of simulation workflow for molecular dynamics and free energy simulations. *M* denotes either 1,2-propanediol, a hydronium ion, or a chloride ion. **(B)** Simulation snapshots of hydronium ion in pure water, 90 wt% DIOX, and 90 wt% DMSO. The hydronium ion is located at the center and only solvent molecules within a 5 Å radius is shown.

We initialized simulation configurations using the protocol schematically depicted in Figure 2A. The initial simulation box containing water and cosolvent (if applicable) had dimensions of (6 nm)^3^ in all simulations and was equilibrated in a *NPT* simulation for 5 ns at *T* = 300 K and *P* = 1 bar with a velocity-rescale thermostat and Berendsen barostat. A single reactant or ion molecule (designated as “M” in Figure 2A) was then added to the system and equilibrated with the same barostat and thermostat for 500 ps. *NPT* production simulations were performed for all systems for 200 ns with a Parrinello-Rahman barostat and Nose-Hoover thermostat; simulations of the reactant, PDO, were performed at *T* = 433.15 K to match the experimental reaction temperature (Mellmer et al., 2018) while simulations of the hydronium/chloride ions were performed at *T* = 300 K. Simulation configurations were output every 10 ps and the final 190 ns of each production trajectory were used for analysis. Simulation analysis was performed using the *MDTraj* library (McGibbon et al., 2015) and analysis tools developed in-house. MD simulations were performed using a leapfrog integrator with a 2-fs time step. Figure 2B shows simulation snapshots of the nearby solvent environment around a hydronium ion in 90 wt% DIOX, 90 wt% DMSO, and pure water.

Each solvation free energy was computed from a series of stochastic dynamics simulations (Figure 2A). Simulations were initialized using an equilibrated solvent system (as described above) with a hydronium or chloride ion added to the system. The total potential of the system was defined as a function of two parameters, λ_*LJ*_ and λ_*elec*_, which scale the LJ and electrostatic potentials between the solute and solvent, as shown in Equation 1:

(1) $$\begin{array}{ll} U\left(\lambda_{LJ},\lambda_{elec}\right) & =U_{M,\;solv}^{LJ}\left(\lambda_{LJ}\right)+\;U_{M,\;solv}^{elec}\left(\lambda_{elec}\right)+U_{M}^{bonded}\\ \; & +U_{M}^{nonbonded}+U_{solv}^{bonded}+U_{solv}^{nonbonded} \end{array}$$

$U_{M,\;solv}^{LJ}$ and $U_{M,\;solv}^{elec}$ are the LJ and electrostatic potentials between solute and solvent, $U_{M}^{bonded}$ and $U_{M}^{nonbonded}$ are intramolecular bonded and non-bonded potentials of the solute, and $U_{solv}^{bonded}$ and $U_{solv}^{nonbonded}$ are the bonded and non-bonded potentials between all solvent molecules (Shivakumar et al., 2010). We performed 17 independent simulations for each solvation free energy: fourteen in which λ_*elec*_ = 0.00 and λ_*LJ*_ = 0.00, 0.00922, 0.04794, 0.11505, 0.260634, 0.31608, 0.43738, 0.56262, 0.68392, 0.79366, 0.88495, 0.95206, 0.99078, or 1.00, and three in which λ_*LJ*_ = 1.00 and λ_*elec*_ = 0.25, 0.75, or 1.00. The LJ coupling parameters represent a 12-point Gaussian sequence, used previously to verify ion model parameters (Horinek et al., 2009; Bonthuis et al., 2016). All free energy simulations used a soft-core LJ potential as described in the Supplementary Section 2.1, Supplementary Material (Beutler et al., 1994). For each simulation, the system was energy minimized with the steepest descent algorithm and equilibrated with a 100 ps *NVT* simulation followed by a 2 ns *NPT* simulation with the Berendsen barostat. An 11 ns *NPT* production simulation was then performed with the Parrinello-Rahman barostat. All simulations were performed at *T* = 300 K and *P* = 1 bar. Energy differences computed between all pairs of windows were collected every 0.2 ps and solvation free energies were computed with the Multistate Bennett Acceptance Ratio (Shirts and Chodera, 2008) method using the python alchemical-analysis tool (Klimovich et al., 2015). The 11 ns of each *NPT* production simulation were split into two 5.5 ns trajectories and treated as two independent trials. All solvation free energy results and error bars are reported as the average and standard deviation of the two trials, respectively. We further calculated three analytical correction terms to account for: (1) finite-size effects due to system interactions with periodic images, (2) the compression free energy for transferring an ion from a 1 atm ideal gas phase to 1 mol/L ideal solution, and (3) the electrostatic energy required to pass through an interfacial potential when the ion transfers from vacuum to bulk solution. These correction terms are included to account for differences between simulation and experiments as described in Supplementary Section 2.2, Supplementary Material.

## Results

### Comparison Between Experimental Reaction Rates and Preferential Exclusion Coefficient

In our previous study of solution-phase acid-catalyzed reactions (Walker et al., 2019), we hypothesized that the transition state is lower in free energy relative to the initial reactant state in mixed-solvent environments due to two reasons: (1) the catalyst is destabilized in bulk solvent relative to a water-enriched local domain near the reactant, leading to a thermodynamic driving force for the transfer of catalytic protons to the local domain, and (2) the transition state is stabilized by water confined within this domain. We then developed a correlative model for experimental reaction rates by quantifying water enrichment in the vicinity of the reactant, supporting the hypothesis for aqueous mixtures of DIOX and GVL. This hypothesis assumes that the hydronium ion catalyst has a higher affinity for water than the cosolvent. However, this assumption may not be accurate for more basic cosolvents, such as DMSO, which can favorably stabilize the acid catalyst in bulk solution (Mellmer et al., 2018). We thus test the validity of this assumption by determining if experimental reaction rates correlate with water enrichment for a model reaction, the Brønsted acid-catalyzed conversion of 1,2-propanediol (PDO) to propanal (Figure 1A), in DIOX and DMSO mixed-solvent environments. These cosolvents represent extremes in polarity: the dielectric constant of DIOX is 2.20, whereas the dielectric constant of DMSO is 48.90 (Fowler et al., 1971).

**Figure 3:**
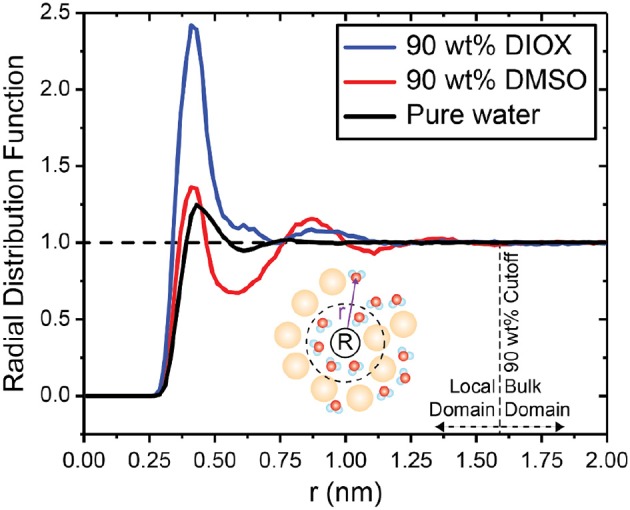
Radial distribution function between the center of mass of PDO and water in 90 wt% DIOX, 90 wt% DMSO, and pure water. Local and bulk domain cutoffs were determined as the value of *r* for which the RDF reaches unity. Bin widths for the RDFs were set to 0.02 nm.

We first analyze the solvent environment around PDO by calculating the radial distribution function (RDF). The RDF quantifies the solvent density, normalized by the bulk solvent density, at a distance *r* away from a central point. Figure 3 shows the RDF between the center of mass of PDO and water for 90 wt% DIOX, 90 wt% DMSO, and pure water. In 90 wt% DIOX, the peak of the RDF is significantly higher than in pure water, indicating that water preferentially partitions to the local solvent domain around PDO in high concentrations of DIOX. Conversely, in 90 wt% DMSO, the first peak of the RDF is almost the same as in pure water and the RDF then drops below unity at ~0.60 nm, indicating the local depletion of water. The diminished water content near PDO in aqueous mixtures of DMSO is due to the cosolvent's high affinity for oxygen groups, resulting in a competition between water and DMSO for the hydroxyl groups of PDO (Vishnyakov et al., 2001). These findings confirm that DMSO and DIOX significantly influence the extent to which the reactant preferentially recruits water to the local domain.

Since RDFs are difficult to compare across different cosolvent concentrations, we previously computed the preferential exclusion coefficient (Γ), a molecular descriptor that quantifies the local domain composition around the reactant (Walker et al., 2019). Γ is defined as the excess number of cosolvent molecules within the local solvent domain of the reactant relative to the bulk solvent domain, computed using Equation 2 (Kang and Smith, 2007; Shulgin and Ruckenstein, 2007; Schneider and Trout, 2009; Shukla and Trout, 2011):

(2) $$\begin{array}{l} \Gamma =-\langle \;n_{C}^{L}-n_{W}^{L}\left(\frac{n_{C}^{B}}{n_{W}^{B}}\right)\rangle \end{array}$$

*n*_*C*_ and *n*_*W*_ denote the number of cosolvent and water molecules, and superscripts *L* and *B* indicate molecules within the local and bulk domains, respectively. We define the boundary between local and bulk solvent domains as the value of *r* at which the RDF reaches unity (Figure 3), which occurs at *r* = 1.59 nm for both solvent systems. Positive Γ values indicate lower concentrations of cosolvent in the local solvent domain of the reactant compared to the bulk solvent domain. Therefore, positive Γ indicates the reactant has a higher affinity for water. Conversely, negative values of Γ indicate that the reactant has a higher affinity for the cosolvent.

We previously found that simulation-derived Γ correlates with experimental reaction rates quantified by the kinetic solvent parameter (σ) defined in Equation (3) (Walker et al., 2019):

(3) $$\begin{array}{l} \sigma_{org,j}^{i}=\left(\frac{k_{org,j}^{i}}{k_{{\text{H}}_{2}O}^{i}}\right) \end{array}$$

$\sigma_{org,j}^{i}$ is the kinetic solvent parameter for the *i*th reaction and the subscript denotes the identity and composition (in *j*th mass fraction) of the organic solvent, $k_{org,j}^{i}$ is the apparent rate constant in aqueous mixtures with the organic phase, and $k_{H_{2}O}^{i}$ is the apparent rate constant in pure water. For simplicity, we denote $\sigma_{org,j}^{i}$ as σ. Positive σ values indicate that the reaction occurs more favorably in aqueous mixtures with organic solvents compared to pure water. Negative σ values indicate the converse. We take experimental reaction rates from Mellmer et al. (2018), which are tabulated in Supplementary Section 3, Supplementary Material.

**Figure 4:**
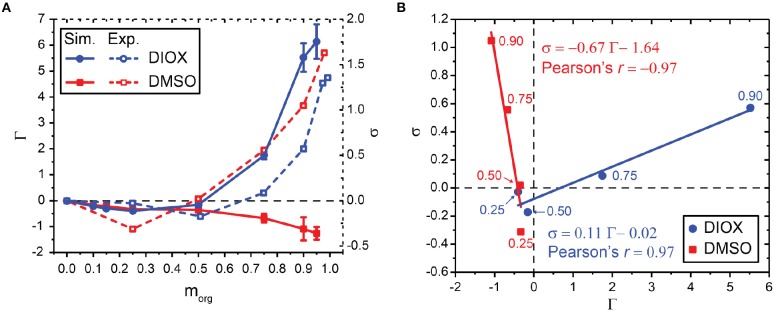
**(A)** Relationship between simulated preferential exclusion coefficient (Γ) and experimentally determined kinetic solvent parameters (σ) for aqueous mixtures of DIOX and DMSO. Experimental values were taken from Mellmer et al. (2018). **(B)** Correlation between Γ and σ for aqueous mixtures of DIOX and DMSO. Data points are labeled with the wt% of the organic solvent. 25, 50, 75, and 90 wt% organic solvent was used to correlate Γ and σ as indicated for each point. The best-fit line is drawn and labeled with the corresponding equation and Pearson's *r*.

Figure 4A compares values of simulation-derived Γ (filled lines) and experimentally measured σ (Mellmer et al., 2018) (dashed lines) for aqueous mixtures of DIOX and DMSO for the PDO dehydration reaction. In each separate mixed-solvent environment, Γ and σ are correlated across the solvent composition range as shown in Figure 4B. We report the Pearson correlation coefficient (Pearson's *r*) as an indicator of linear correlation: values close to 1 indicate total positive linear correlation, values close to −1 indicate total negative linear correlation, and values close to 0 indicate no linear correlation. We find *r* = 0.97 for aqueous mixtures of DIOX, indicating strong positive linear correlation. However, we find *r* = −0.97 for aqueous mixtures of DMSO, indicating strong negative correlation and that the depletion of water around PDO in DMSO mixtures still leads to enhanced reaction kinetics. These results indicate that in either solvent system the preferential exclusion coefficient can predict reaction kinetics; however, the negative correlation between Γ and σ in DMSO suggests that increased reaction rates are not due to water enrichment. This finding suggests that the assumption that the acid catalyst preferentially partitions to water-enriched regions of the system is not valid for all cosolvents and must be revised to derive a correlative model for reaction rates that can be broadly applied to any cosolvent of interest.

### Solvation Free Energy of the Hydronium Ion in Pure Solvent Systems

Previous studies have found that the hydronium ion is more stable in DMSO than water based on lower solvation free energies (Kelly et al., 2007). Since our simulations show that PDO preferentially interacts with DMSO rather than water and PDO reaction rates are increased in high concentrations of DMSO, the reactant/proton complex may be stabilized in the organic phase compared to the water phase, leading to increased reaction rates. Therefore, we hypothesize that the solvation free energy of the hydronium ion catalyst in the organic solvent can be used to classify the preference for the catalyst for either water or organic phase and develop an updated correlative model between Γ and σ for a range of cosolvents.

**Figure 5:**
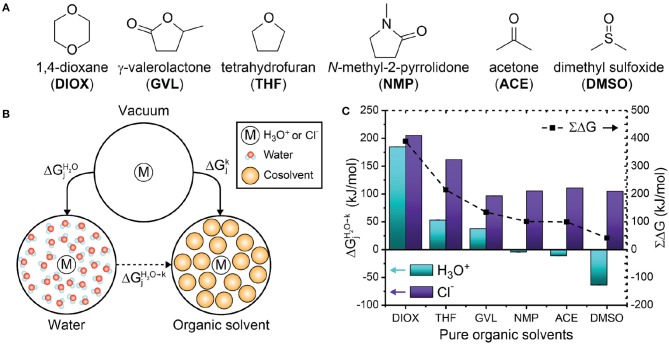
**(A)** Chemical structures of the organic solvents used in this study. **(B)** Thermodynamic cycle used to compute the free energy for transferring a hydronium or chloride ion from pure water to pure organic solvent. $\Delta G_{j}^{k}$ and $\Delta G_{j}^{H_{2}O}$ are solvation free energies while $\Delta G_{i}^{H_{2}O\to k}$ is the transfer free energy computed from Equation 4. **(C)** Transfer free energies for six pure organic solvents. Cyan and purple bars indicate hydronium (${\text{H}}_{3}{\text{O}}^{+}$) and chloride (Cl^−^) ion transfer free energies, respectively. Dashed lines indicate the sum of the transfer energies. Error bars were computed from the standard deviation of two trials; the error is <1 kJ/mol and is not visible in the plot. The error is tabulated in Supplementary Table 4.

We calculated the solvation free energy of the hydronium ion in six organic, polar aprotic solvents (Figure 5A) and performed the same calculations for a chloride ion to determine the solvation free energy for a conjugate base. We selected polar aprotic solvents due to their relevance to acid-catalyzed biomass conversion processes, in which inclusion of these solvents has been found to enhance reaction performance (Mellmer et al., 2014b, 2018; Walker et al., 2019). To test the simulation approach, we first calculated solvation free energies in pure water as −465.1 kJ/mol [experimentally measured as −453.2 kJ/mol (Pliego, 2003)] for the hydronium ion and −286.4 kJ/mol [experimentally measured as −304.6 kJ/mol (Pliego and Riveros, 2000)] for the chloride ion; their sum of −751.5 kJ/mol is comparable to the estimated experimental value of −757.8 kJ/mol (Pliego and Riveros, 2000; Pliego, 2003). The experimental values reproduced from Pliego (2003) and Pliego and Riveros (2000) are modified to include the 7.9 kJ/mol correction term associated with transferring an ion from 1 atm ideal gas phase to 1 mol/L ideal solution to compare with our results (Supplementary Section 2.2, Supplementary Material). The relative differences between solvation free energies are more important than absolute values (Horinek et al., 2009) for inferring the behavior of the ions in different solvents; therefore, we focus on relative transfer free energies between pure water and organic solvents.

We computed the free energy of transferring the hydronium or chloride ion from water to solvent systems with organic solvents using Equation 4 (schematically illustrated in Figure 5B):

(4) $$\begin{array}{l} \Delta G_{j}^{H_{2}O\to k}=\Delta G_{j}^{k}-\Delta G_{j}^{H_{2}O} \end{array}$$

*k* denotes the solvent system of interest and *j* denotes either a hydronium or chloride ion. A negative value of $\Delta G_{j}^{H_{2}O\to k}$ indicates that the ion is thermodynamically stabilized in the *k*th solvent system compared to pure water. Figure 5C shows $\Delta G_{j}^{H_{2}O\to k}$ of the hydronium and chloride ions in each pure solvent. For the hydronium ion (cyan bars), $\Delta G_{H_{3}O^{+}}^{H_{2}O\to k}$ is positive for DIOX, GVL, and THF, indicating that the hydronium ion is unfavorable in these solvents. These results support our prior assumption that the hydronium ion prefers water rather than the organic phase in these solvents, allowing us to correlate the formation of water-enriched local domains to reaction kinetics (Walker et al., 2019). Conversely, $\Delta G_{H_{3}O^{+}}^{H_{2}O\to k}$ is negative for NMP, ACE and DMSO, indicating that the hydronium ion is favorable in these solvents. Notably, these solvents are more basic than water (Fawcett, 1993) based on several solvent scales (e.g., *B* parameter of Koppel and Palm, 1972, or Kamlet-Taft β scale, Kamlet and Taft, 1976; Fawcett, 1993) (discussed below and in Table 1). The negative free energy for transferring a hydronium ion from water to DMSO agrees with prior results (Kelly et al., 2007; Mellmer et al., 2018) and supports the hypothesis that the sign of this free energy change determines the relationship between Γ and σ for DIOX and DMSO mixtures (Figure 4). In a similar fashion, we suspect that ACE and DMSO would exhibit similar solvent effects.

**Table 1:** Dielectric constants and Kamlet-Taft parameters (α, β, π^*^) for pure solvents, tabulated according to decreasing transfer free energy of a hydronium ion ($\Delta G_{H_{3}O^{+}}^{k\to H_{2}O}$) in these solvents. ∑Δ*G* was computed with Equation 5.

		**Kamlet-taft Parameters**				
**Solvent**	**Dielectric constant^**a**^**	**α^**b**^**	**β^**b**^**	π^*^^c^	$\Delta G_{H_{3}O^{+}}^{k\to H_{2}O}$	**∑*ΔG***
DIOX	2.20	0.00	0.37	0.49	184.6	389.85
THF	7.40	0.00	0.55	0.55	53.0	214.91
GVL	36.47	0.00	0.60	0.83	37.8	134.36
Water	78.50	1.17	0.47	1.14	0.0	0.00
NMP	32.16	0.00	0.77	0.92	−4.3	101.25
ACE	20.70	0.08	0.43	0.62	−11.2	99.55
DMSO	48.90	0.00	0.76	1.00	−63.5	41.69

All solvation free energies are in units of kJ/mol.

a Values are from Fowler et al. (1971), except for GVL (Wohlfarth, 2015), and NMP (Uosaki et al., 1996).

b Values from Marcus (1993), except for GVL (Jessop et al., 2012).

c *Values from Laurence et al. (1994), except for GVL (Jessop et al., 2012), and water (Buhvestov et al., 1998)*.

### Solvation Free Energy of the Chloride Ion in Pure Solvent Systems

While the solvation free energy of the hydronium ion alone quantifies catalyst stability, the effect of the solvent on acid dissociation equilibrium depends on the solvation free energy of the hydronium ion and its conjugate base (i.e., the chloride ion). Figure 5C shows that $\Delta G_{Cl^{-}}^{H_{2}O\to k}$ is positive for all pure organic solvent systems (purple bars), indicating that the chloride ion thermodynamically prefers water over each of these solvents. Furthermore, the solvation free energies for the chloride ion do not vary significantly for the different organic solvents, with the exception of DIOX and THF. The difference in the solvation of the hydronium and chloride ions is likely due to differences in hydrogen bonding capabilities: the hydronium ion can donate and accept hydrogen bonds while the chloride ion can only accept hydrogen bonds. Since water is the only solvent in this study that can donate hydrogen bonds, it is expected that the chloride ion is most stable in water.

The effect of the solvent on the solvation free energies of the hydronium and chloride ions relative to their solvation free energies in water is quantified via the term ∑Δ*G*, which we define in Equation 5 as:

(5) $$\begin{array}{l} \sum \Delta G=\sum \Delta G_{H_{3}O^{+}Cl^{-}}^{k\to H_{2}O}=\Delta G_{H_{3}O^{+}}^{H_{2}O\to k}+\;\Delta G_{Cl^{-}}^{H_{2}O\to k} \end{array}$$

We expect that positive values of ∑Δ*G* would reduce acid dissociation relative to pure water due to the decreased stability of the dissociated ions in the pure organic solvent. Figure 5C shows that ∑Δ*G* is positive for each organic solvent (black dashed lines). This result suggests that all of the polar aprotic solvents would decrease acid dissociation, leading to the reduced catalyst availability associated with weak acids based on experiments (Mellmer et al., 2014b). The sign of ∑Δ*G* is largely dictated by the solvation free energies of the chloride ion, indicating that the selection of the conjugate base is important for acid disassociation (Mellmer et al., 2014b), although the choice of conjugate base would not affect the solvation free energies of the hydronium ion itself.

### Relationship Between Solvation Free Energies and Solvent Parameters

Given the computational expense of free energy calculations, we next sought to relate the transfer free energy results ($\Delta G_{H_{3}O^{+}}^{k\to H_{2}O}$ and ∑Δ*G*) to tabulated solvent properties to determine if these properties could accelerate solvent screening. Table 1 compares transfer free energy values to solvent dielectric constants and Kamlet-Taft parameters (α, β, π^*^). We use the dielectric constant to quantify the polarizability of the solvents and the Kamlet-Taft parameters to quantify hydrogen-bond donating ability (acidity, α), hydrogen-bond accepting ability (basicity, β), and polarity/polarizability (π^*^) (Kamlet and Taft, 1976; Taft and Kamlet, 1976; Kamlet et al., 1977, 1983). Each of the Kamlet-Taft parameters are scaled from 0 to 1 based on two reference solvents. For instance, π^*^ uses cyclohexane and DMSO as a reference for 0 and 1, respectively (Kamlet et al., 1977; Laurence et al., 1994). We expect that the stability of a hydronium ion can be influenced by the polarity of the solvent; however, neither dielectric constant nor π^*^ quantitatively correlate with $\Delta G_{H_{3}O^{+}}^{k\to H_{2}O}$ or ∑Δ*G*. Furthermore, basicity is expected to be an important metric of whether a hydronium ion is favored in a solvent environment, with larger β values indicating more basic solvents that would favorably solvate the acidic hydronium ion, but there is no clear correlation between β and the free energy results. We also do not find a correlation between α and the free energy results, which is expected since acidity does not directly relate to the stability of a hydronium ion in a solvent system.

These data suggest that typical solvent-specific parameters cannot easily describe the interplay of solute-solvent interactions and solvent reorganization that dictate the measured transfer free energies. We further computed the RDF between the hydronium ion and pure solvents (Supplementary Figure 7, Supplementary Material) to determine if solvent structure correlated with the transfer free energies, but we do not find a clear trend to explain the results found in Figure 5C. This data thus suggests that the free energy calculations are providing new information that can be used to predict the preference of the hydronium ion for either water or an organic solvent and quantify the effect of solvent composition on acid dissociation. It is also possible that the MD workflow is insufficiently accurate to predict these values, particularly given the classical model of the hydronium ion. However, the good agreement between the calculated solvation free energies of the hydronium and chloride ions in water with experimental data suggests that the model is reasonable. We also emphasize that DIOX, THF, and GVL have positive values of $\Delta G_{H_{3}O^{+}}^{k\to H_{2}O}$ and lead to increased reaction rates in mixed-solvent systems when water is enriched near the reactant, while DMSO has a negative value of $\Delta G_{H_{3}O^{+}}^{k\to H_{2}O}$ and leads to increased reaction rates in mixed-solvent systems when the cosolvent is enriched near the reactant. The distinct behavior of these cosolvents mirrors the difference in the sign of the calculated transfer free energies, suggesting that the transfer free energies are correctly capturing differences in the preference of the hydronium ion for bulk organic solvent.

### Transfer Free Energies of the Hydronium and Chloride Ion in Mixed-Solvent Systems

**Figure 6:**
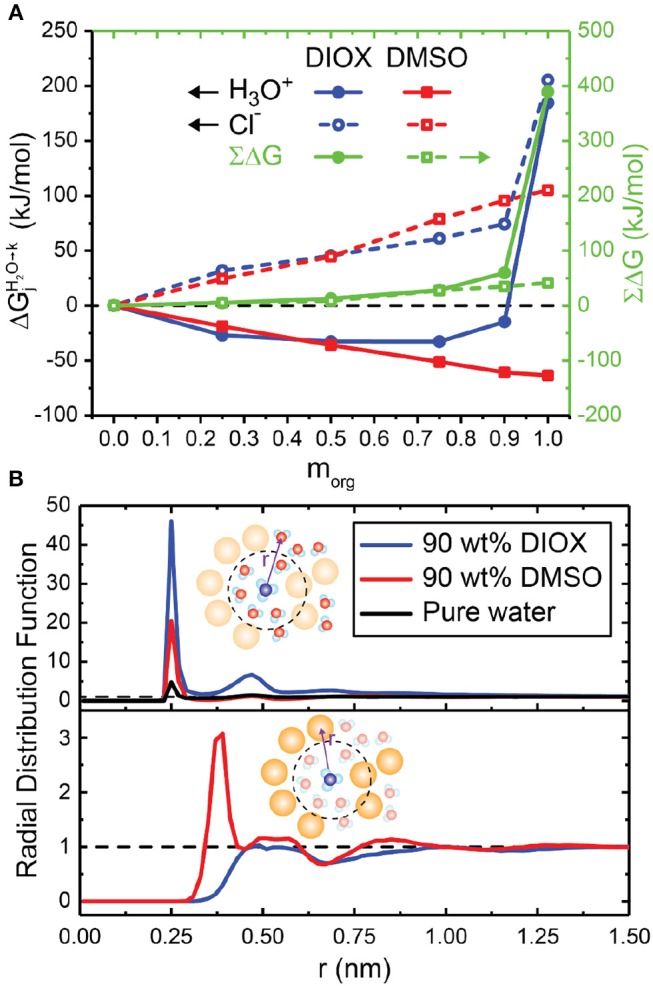
**(A)** Transfer free energies for transferring hydronium (${\text{H}}_{3}{\text{O}}^{+}$, filled lines) and chloride (Cl^−^, dashed lines) ions from pure water to aqueous mixtures of dioxane (DIOX, blue lines) and dimethyl sulfoxide (DMSO, red lines). The sums of the transfer free energies (∑Δ*G*) are shown as green lines. Error bars are not shown; they range from 0 to 2.5 kJ/mol when averaging two trials and tabulated in Supplementary Table 5. **(B)** Radial distribution function (RDF) between the center of mass of the hydronium ion to water (top) and the organic solvent (bottom) in 90 wt% DIOX, 90 wt% DMSO, and pure water. Bin widths for the RDFs were set to 0.02 nm.

Figure 6A shows the free energies for transferring either a hydronium or chloride ion to aqueous mixtures of DMSO and DIOX from pure water; these solvents represent extrema of low and high affinity cosolvents for the hydronium ion. In aqueous mixtures of DMSO, Figure 6A shows a monotonic decrease in the hydronium ion transfer free energy (i.e., an increase in hydronium ion stability relative to pure water) as the mass fraction of the organic phase increases. Since the free energy calculations in pure organic solvents (Figure 5C) show that pure DMSO stabilizes the hydronium ion more than water, these results agree with the expectation that increasing concentrations of DMSO results in improved stability of the hydronium ion. Figure 6B shows RDFs between the hydronium ion and both water and DMSO in 90 wt% DMSO. The peak of the ion-water RDF in 90 wt% DMSO is higher than in pure water, showing a local enrichment of water around the ion; however, there is also a cosolvent peak at ~0.38 nm, showing an enrichment in DMSO. Therefore, water and DMSO both favorable solvate the hydronium ion (visually shown in Figure 2C), leading to its increased stability relative to pure water. These results are consistent with experimental trends that find that increasing the concentration of DMSO monotonically increases basicity (Catalán et al., 2001). The results further suggest that there should be a driving force to partition the hydronium ion to regions of the solvent system that have the highest concentration of DMSO to reduce its free energy to the greatest extent, agreeing with the hypothesis that local enrichment of DMSO around a reactant leads to an increase in acid-catalyzed reaction rates.

In aqueous mixtures of DIOX, Figure 6A shows a non-monotonic trend in the hydronium ion transfer free energy as the mass fraction of the organic phase increases. The transfer free energy is negative for all mixed compositions indicating that the hydronium ion is more stable than in either pure solvent. In the RDFs presented in Figure 6B, the peak of the ion-water RDF in 90 wt% DIOX is almost 10-fold larger than the peak in pure water, indicating a significant enrichment of water around the hydronium ion (visually shown in Figure 2B). In addition, the cosolvent RDF (Figure 6B, bottom) shows that DIOX is depleted near the hydronium ion up to distances of about 1 nm. These results together indicate that the hydronium ion nucleates a local domain of water molecules confined within the vicinity of the ion by the surrounding cosolvent. We attribute the decreased free energy of the hydronium ion in the mixed-solvent environment to the formation of this domain, which effectively sequesters water molecules to eliminate unfavorable water-cosolvent interactions that are not present in either pure solvent. Surprisingly, this data suggests that there should not be a driving force for hydronium ions to partition from bulk mixed-solvent environments to water-enriched domains near hydrophilic reactants as previously hypothesized because the solvation free energy of the ion in pure water is higher than in the mixed-solvent environment. This finding suggests that the stabilization of the charged transition state by confined water molecules in the water-enriched local domain might be the dominant factor leading to increased reaction rates. However, these calculations omit explicit modeling of the reactant, which could affect partitioning thermodynamics.

Finally, Figure 6A shows that the chloride ion is not favored in any mixed-solvent composition, resulting in positive $\Delta G_{j}^{H_{2}O\to k}$ and ∑Δ*G* values for all DIOX and DMSO mass fractions. These data again indicate that acid dissociation is preferred in pure water rather than any mixed-solvent environment and thus weak acids are less likely to dissociate, diminishing reaction performance.

## Discussion

### Screening Solvent Properties Using a Classical Hydronium Ion Model

Recent studies of acid-catalyzed biomass conversion reactions have illustrated that the stability of the hydronium ion catalyst in various mixed-solvent environments can dramatically affect reaction rates (Mellmer et al., 2014a,b, 2018; Shuai and Luterbacher, 2016; He et al., 2017; Walker et al., 2018, 2019; Varghese and Mushrif, 2019). Computational tools have been developed to study the hydronium ion in different solvent systems (Tawa et al., 1998; Bonthuis et al., 2016; Varghese and Mushrif, 2019), with *ab initio* molecular dynamics emerging as a powerful method to study interactions between the hydronium ion and solvent molecules due to the method's accuracy and ability to capture quantum mechanical effects (Tuckerman et al., 1994, 1995a,b, 1997; Sagnella et al., 1996; Marx et al., 1999; Morrone and Tuckerman, 2002; Izvekov and Voth, 2005; Marx, 2006). However, *ab initio* simulations are computationally expensive and difficult to expand across multiple solvent systems. Therefore, we used a classical hydronium ion model (Bonthuis et al., 2016) to compute the stability of the hydronium ion by measuring its solvation free energy in solvent systems with organic, polar aprotic solvents. Our findings suggest that the hydronium ion is unfavorable in DIOX, THF, and GVL solvents but favorable in NMP, ACE, and DMSO solvents (Figure 5C). These results can classify whether a solvent favorably facilitates a hydronium ion to help determine which phase the acid catalyst prefers in mixed-solvent systems. Furthermore, we could not identify a tabulated cosolvent-specific descriptor (e.g., dielectric constant, Kamlet-Taft parameters) that correlates with the hydronium ion solvation free energies, although the solvation free energies qualitatively capture features of solvent scales, such as the large distinction between DIOX and DMSO solvents. The lack of correlation suggests that the solvation free energy calculated from a MD simulation may provide unique information on proton-solvent interactions and can act as a cosolvent-specific descriptor for the stability of the acid catalyst.

In mixed-solvent environments, the solvation structure around the hydronium ion show that water-enriched local domains are formed, analogous to water-enrichment around hydrophilic reactants (Mellmer et al., 2018; Walker et al., 2019), but the magnitude of enrichment is dependent on the choice of organic solvent. DMSO molecules compete with water for binding sites around the hydronium ion, whereas DIOX molecules are depleted around the hydronium ion. The hydronium ion solvation free energies in aqueous mixtures of DIOX suggest that small amounts of water can stabilize the hydronium ion to a greater degree than pure water or DIOX. This stabilization originates from the hydronium ion being confined by water, a solvent environment also found in water-enriched local domains formed by hydrophilic reactants. This finding suggests that stabilization of charged transition states by confined water in mixed-solvent environments may contribute to the increased reaction rates observed experimentally.

In all solvent environments studied, the sum of the transfer free energies of the hydronium and chloride ions from water was positive. This result indicates that non-aqueous environments tend to suppress acid dissociation, leading to lower catalyst availability for weak acids that translates to lower reaction rates (Mellmer et al., 2014b). However, in this work we only studied the solvation free energy of a chloride ion conjugate base, and thus investigating the effect of alternative conjugate bases on acid dissociation could yield different effects on acid dissociation. For example, triflic acid is known to readily disassociate even in high concentrations of DMSO (Mellmer et al., 2018). Future work will thus extend the framework developed here to further screen conjugate bases to determine the effect on acid dissociation, enabling the incorporation of these values into correlative models for reaction optimization.

### Incorporation of Hydronium Ion Stability Into the Correlative Model of Reaction Rates

Figure 4 showed that the acid-catalyzed dehydration of PDO depends on the choice of cosolvent, with the experimentally measured kinetic solvent parameter (σ) correlating with the simulation-derived preferential exclusion parameter (Γ) in aqueous mixtures of DIOX and DMSO. This correlation is based on the physical understanding that catalytic hydronium ions preferentially partition to the water-enriched local domain around the reactant, increasing reaction performance and leading to a positive correlation between σ and Γ (Figure 4B). However, the correlation between σ and Γ is negative in DMSO, a solvent for which water depletion around the reactant is observed while reaction rates still increase. We hypothesized that the negative correlation may be because the hydronium ion preferentially interacts with DMSO rather than water and thus partitions to the water-depleted, DMSO-enriched local domain. This hypothesis is supported by the negative free energy for transferring a hydronium ion from water to DMSO as shown in Figure 5B. Thus, the correlation between Γ and σ must be adjusted to account for the stability of the hydronium ion in the local domain.

We include the sign of the hydronium ion transfer free energy between pure organic solvent to water, $\Delta G_{H_{3}O^{+}}^{H_{2}O\to pure\;org.}$, as a correction term in the preferential exclusion coefficient (Γ′) by using Equation 6:

(6) $$\begin{array}{l} \Gamma^{\prime }=\Gamma^{*}\text{sign}\left(\Delta G_{H_{3}O^{+}}^{H_{2}O\to pure\;org.}\right) \end{array}$$

**Figure 7:**
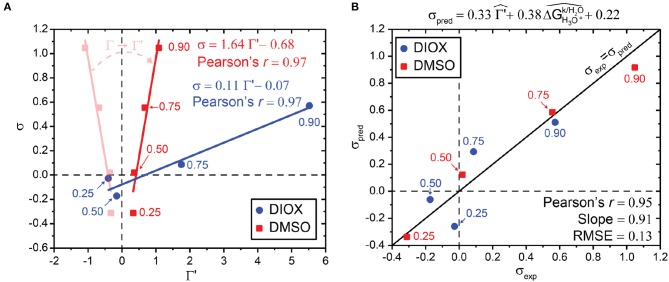
**(A)** Correlation between simulated preferential exclusion coefficient with solvation free energy correction term (Γ′), as expressed in Equation 7, and experimentally determined kinetic solvent parameters (σ) for aqueous mixtures of DIOX and DMSO. Experimental values were taken from Mellmer et al. (2018). Transparent red points and lines show how Γ relates to Γ′. **(B)** Parity plot between predicted kinetic solvent parameter (σ_*pred*_) and experimental kinetic solvent parameter (σ_*exp*_) using results from aqueous mixtures of DIOX and DMSO. The correlative model is based on Equation 9 as shown above the plot. Data points are labeled with the wt% of the organic solvent.

Equation 6 ensures that Γ′ and σ are positively correlated for aqueous mixtures of DMSO as shown in Figure 7A. We interpret Γ′ as quantifying the enrichment of the solvent (water or cosolvent) that preferentially stabilizes the hydronium ion, such that larger values of Γ′ suggest higher catalyst availability in the local domain of the reactant. Since Γ′ and σ are positively correlated for both aqueous mixtures of DIOX and DMSO, we can then write a correlative model for σ_*pred*_ that bridges these distinct solvents using Equation 7:

(7) $$\begin{array}{l} \sigma_{pred}=A\left(\Gamma^{\prime }\right) \end{array}$$

*A* is a constant. Supplementary Figure 8 shows the correlation between σ_*pred*_ and σ_exp_ when combining results from both DIOX-water and DMSO-water mixtures, resulting in a best-fit slope of 0.25 (ideally this value should be unity), and a root-mean-square error (RMSE) between predicted and experimental values of 0.39. Similar to our previous work (Walker et al., 2019), we next explored the use of multiple descriptors in combination to improve this correlation. In particular, we define $\Delta G_{H_{3}O^{+}}^{k/H_{2}O\;}$ as the ratio of the solvation free energy of the hydronium ion in the *k*th solvent system ($\Delta G_{H_{3}O^{+}}^{k\;}$) to the solvation free energy of hydronium ion in pure water ($\Delta G_{H_{3}O^{+}}^{H_{2}O})$.

(8) $$\begin{array}{l} \Delta G_{H_{3}O^{+}}^{k/H_{2}O\;}=\frac{\Delta G_{H_{3}O^{+}}^{k\;}}{\Delta G_{H_{3}O^{+}}^{H_{2}O}} \end{array}$$

Since acid-catalyzed reactions generally form a charged transition state after protonation of the reactant (Figure 1B), we interpret $\Delta G_{H_{3}O^{+}}^{k/H_{2}O\;}$ as a unitless metric that estimates transition state stability in mixed-solvent systems compared to pure water. In our prior work, the reactant-water hydrogen bonding lifetime was identified as a descriptor that quantified transition state stability (Walker et al., 2019); here, $\Delta G_{H_{3}O^{+}}^{k/H_{2}O\;}$generalizes this prior finding to account for non-hydrogen bonding interactions. Supplementary Figure 9 shows $\Delta G_{H_{3}O^{+}}^{k/H_{2}O\;}$ as a function of solvent composition for aqueous mixtures of DIOX and DMSO. For these cosolvents, $\Delta G_{H_{3}O^{+}}^{k/H_{2}O\;}$ is greater than unity.

We combined Γ′ and $\Delta G_{H_{3}O^{+}}^{k/H_{2}O\;}$ using the multilinear regression model shown in Equation 9, where *A*, *B*, and *C* are coefficients. To enable comparison between the coefficients, we standardized Γ′ and $\Delta G_{H_{3}O^{+}}^{k/H_{2}O}$ by subtracting their mean and dividing by their standard deviations as described in Supplementary Section 5.2. All standardized variables are denoted by a hat accent.

(9) $$\begin{array}{l} \sigma_{pred}=A\left(\hat{\Gamma }\prime \right)+B\left(\hat{\Delta {\text{G}}_{H_{3}O^{+}}^{k/H_{2}O}}\right)+C \end{array}$$

Figure 7B shows the correlation between σ_*pred*_ and σ_exp_ when using Equation 9 and combining results from both DIOX-water and DMSO-water mixtures. The best-fit slope is 0.91, close to the ideal value of unity, and the RMSE between predicted and experimental values is 0.13, which is comparable to experimental error (Walker et al., 2019). Furthermore, the coefficients for $\hat{\Gamma }\prime$ and $\Delta G_{H_{3}O^{+}}^{k/H_{2}O}$ are comparable (0.33 vs. 0.38), suggesting that solvent enrichment around the reactant that favors the hydronium ion catalyst and the transition state stability are important variables for the prediction of acid-catalyzed reaction kinetics. We thus find that including information on the hydronium ion solvation free energy in an organic solvent can improve the correlation between Γ and σ when considering aqueous mixtures with different polar aprotic cosolvents. We note that additional solvent-specific descriptors (e.g., hydrogen bonding between water and the organic phase, etc.) may improve the correlation between different solvent systems and is a subject of future research.

## Conclusions

We performed classical molecular dynamics simulations and solvation free energy calculations to quantify the stability of hydronium and chloride ions in six organic, polar aprotic solvents. We found that the hydronium ion is favorably solvated in pure NMP, ACE, and DMSO solvents, but unfavorably solvated in pure DIOX, THF, and GVL solvents. In mixed-solvent environments, the inclusion of water with DIOX stabilizes the hydronium ion more than their pure solvent counterparts. We attribute this increased stabilization to the formation of water-enriched local solvent domains around the hydronium ion. In aqueous mixtures of DMSO, the hydronium ion is further stabilized with increasing concentration of the organic phase. Conversely, the chloride ion is destabilized in all pure organic solvents and mixed solvent systems, inhibiting acid dissociation. By quantifying the stability of the hydronium ion in organic solvents, we obtained a new cosolvent-specific descriptor that quantifies acid catalyst stability. We incorporated this descriptor into a correlative model for 1,2-propanediol dehydration reaction rates to demonstrate that the solvation free energy results can be used to bridge reaction rate predictions across different cosolvent systems. Incorporating information about the acid catalyst stability in different solvent mixtures represents an important step toward the rational design of mixed-solvent environments for acid-catalyzed reaction schemes and has the potential to alleviate time-intensive experimentation that accompanies the optimization of biomass conversion reactions for maximum productivity.

## Data Availability

All datasets generated for this study are included in the manuscript and/or the Supplementary Files.

## Author Contributions

AC designed, performed, and analyzed the molecular dynamics simulations. AC and RV conceived of the simulations, interpreted the results, and wrote the manuscript.

### Conflict of Interest Statement

The authors declare that the research was conducted in the absence of any commercial or financial relationships that could be construed as a potential conflict of interest.
