# Supplementary material for: Quantifying the Stability of the Hydronium Ion in Organic Solvents With Molecular Dynamics Simulations
Source: Front Chem. 2019 Jun 19;7:439. doi: 10.3389/fchem.2019.00439 (PMC6594219; doi:10.3389/fchem.2019.00439)
Supplement: Supplementary file 1 [file Table_1.DOCX]

Supplementary Material

# Hydronium ion force field parameters

The hydronium ion model developed by Bonthuis *et al.* was originally implemented using the SHAKE constraint algorithm [1]. To facilitate simulations of large mixed-solvent systems in parallel, we adjusted the hydronium ion model to use the LINCS algorithm, which is three to four times faster than the SHAKE algorithm [2]. In the original model, the authors constrained the O-H bond length to 0.98 A and H-H bond length to 0.1619 nm. This approach leads to numerical stability problems when using LINCS. Instead, we eliminated the H-H bond constraint and added a harmonic restraint to maintain a 111.4° H-O-H bond angle. We varied the spring constant for the angle restraint and computed the solvation free energy of a hydronium ion in pure water to gauge the accuracy of the model. Supplementary Figure 1 shows the molecular dynamics (MD) simulated solvation free energies using different spring constants. We found that increasing spring constants better match the results found from the SHAKE algorithm. We concluded that an angle force constant of 2,000.0 kJ/mol/rad^2^ is sufficient to perform MD simulations with the LINCS algorithm and a 2-fs time step, particularly since we focus on differences in solvation free energies in the main text. Force constants larger than 2,000 kJ/mol/rad^2^ led to numerical instabilities, necessitating a 1-fs timestep that drastically reduced simulation performance. Force field parameters for the hydronium ion model are summarized in Supplementary Table 1.


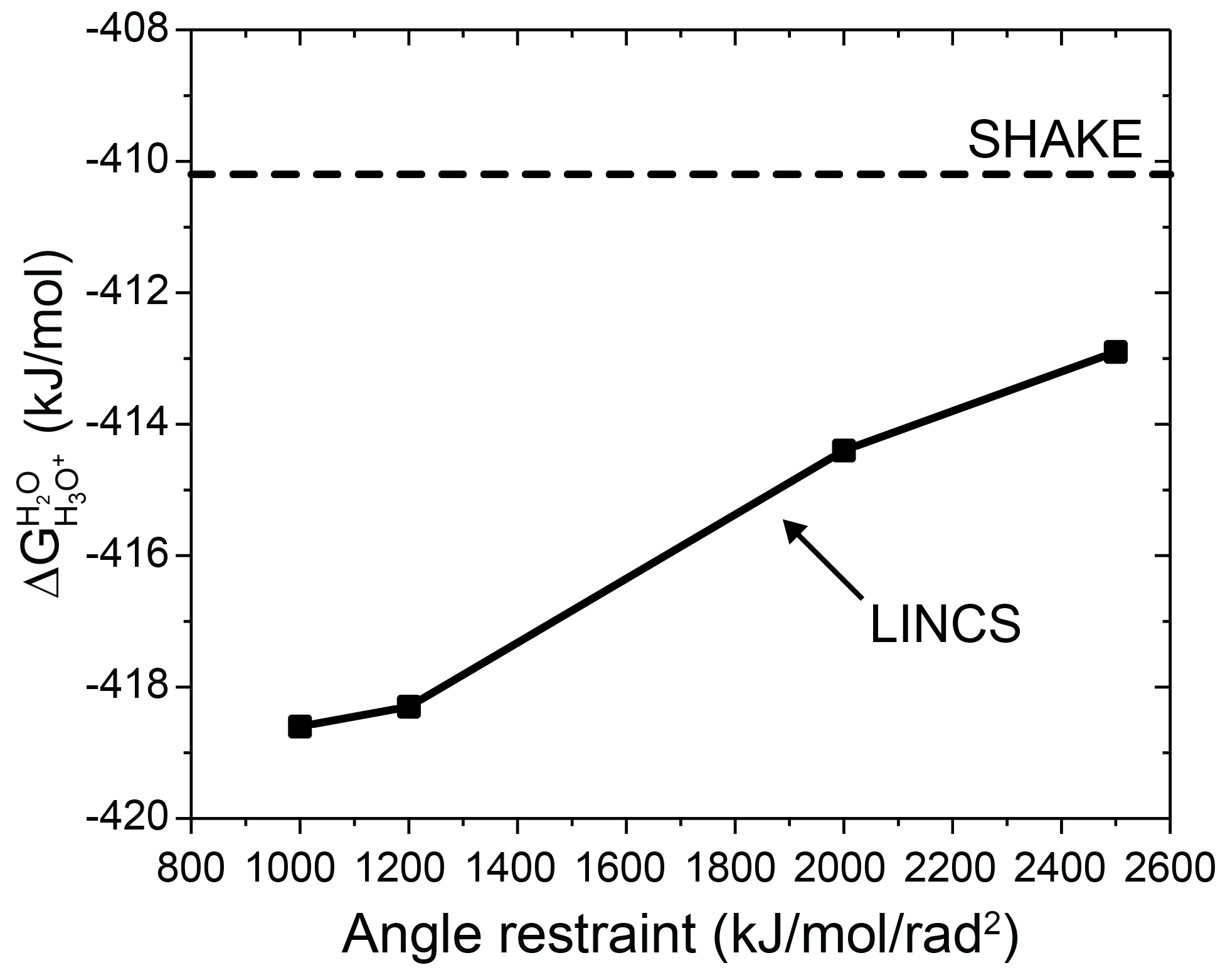


**Supplementary Figure 1.** Solvation free energies of a hydronium ion in pure water for systems using the LINCS constraint for bond constraints and an angle restraint to maintain the H-O-H bond angle. The dashed line shows the free energy when using the SHAKE algorithm and constraint methodology specified in Ref. [1]. Solvation free energies were computed with shorter 5 ns *NPT* simulations compared to the 11 ns *NPT* simulations in the main text. These solvation free energies do not take into account any correction terms as described in Section 2.2.

**Supplementary Table 1.** Force field parameters for the hydronium ion. σ and ε are Lennard-Jones parameters taken from Ref. [1].

| Atomtypes | | | | |
| --- | --- | --- | --- | --- |
| Atoms | Charge | Atomic mass (amu) | σ (nm) | ε (kJ/mol) |
| O | -1.4 | 15.9994 | 0.31 | 0.8 |
| H | 0.8 | 1.008 | 0 | 0 |
|  |  |  |  |  |
|  | Bonds | | |  |
|  | Bonds | Length (nm) | Force constant [kJ/mol/nm^2^] |  |
|  | O-H | 0.098 | 345,000 |  |
|  |  |  |  |  |
|  | Angles | | |  |
|  | Angles | Angle (degrees) | Force constant [kJ/mol/rad^2^] |  |
|  | H-O-H | 111.4 | 2,000 |  |

# Additional simulation details for free energy calculations

## Soft-core potentials for van der Waals interactions

In GROMACS, soft-core potentials are typically used in solvation free energy calculations to avoid numerical stability and convergence issues when $r_{ij}$, the distance between species $i$ and $j$, approaches zero [3], described in detail elsewhere [1, 4]. For this work, all free energy simulations used soft-core parameter $\sigma_{sc}=0.3 \mathrm{nm}$, $\alpha=0.5$, and an exponent $p=1$ utilizing the standard GROMACS soft-core Lennard-Jones potential.

## Correction terms

We include three correction terms to account for differences between simulations and experiments. Corrections for finite-size effects, or effects due to system interactions with periodic images, are known to be especially important when the net charge of the system changes during a free energy calculation. Therefore, analytical solutions have been proposed to correct for the influence of the system size and improve comparison with experiments [5]. The free energy of solvation ($\Delta G_{solv}$) is computed from Equation 1:

|  | $\Delta G_{solv}=\Delta G_{sim}+\Delta G_{fs}+\Delta G_{press}+\Delta G_{surf}$ | (1) |
| --- | --- | --- |

where $\Delta G_{sim}$ is the simulated solvation free energy, $\Delta G_{fs}$ is the free energy correction factor for finite-size effects, $\Delta G_{press}$ is the free energy correction factor for compression, and $\Delta G_{surf}$ is the free energy correction for ion transfer across a vapor-liquid interface.

$\Delta G_{fs}$ accounts for a monovalent ion’s interaction with its periodic images due to long-range electrostatic interactions. This correction takes into the interaction energy of the ion with its own periodic image in vacuum and in solvent, summarized by Equation 2 [1, 5]:

|  | $\Delta G_{fs}=\frac{e^{2}(\epsilon-1)}{6\varepsilon\varepsilon_{0}L}\left[ \left( \frac{r_{a}}{L} \right)^{2}-\frac{4\pi}{15}\left( \frac{r_{a}}{L} \right)^{5} \right] -\frac{e^{2}\xi}{8\pi\varepsilon\varepsilon_{0}L}$ | (2) |
| --- | --- | --- |

where $\epsilon$ is the dielectric constant, $L$ is the ensemble-average box length, $r_{a}$ is the ion radius (0.100 nm and 0.181 nm for the hydronium [6] and chloride [7] ions, respectively), $\varepsilon_{0}$ is the permittivity of vacuum ($\varepsilon_{0}=5.727\times{10}^{-4}\frac{e^{2}mol}{kJ\cdot nm}$), $\xi$ is the prefactor Wigner energy per particle on a cubic lattice ($\xi=-2.837297$) and $e$ is the elementary charge ($e=1.602176487\times{10}^{-19}C$). Note that Equation 2 is not precisely applicable for the hydronium ion due to partial charges and difficulty in computing $r_{a}$, but it provides an estimate for correcting interactions with periodic images. We compute the dielectric constant of each solvent system in a separate MD simulation (Section 2.2.2). For instance, we computed $\varepsilon=72$ for pure water which matches the dielectric constant of bulk SPC/E water ($\varepsilon=71$) [8]. $\Delta G_{fs}$ is generally between 0-2 kJ/mol for high dielectric solvents like water (~0.7-0.8 kJ/mol), but can become significant for low dielectric constant solvents like dioxane (~36 kJ/mol).

$\Delta G_{press}$ accounts for the hypothetical transfer of a 1 atm ideal gas phase into a 1 mol/L ideal solution, resulting in a compression free energy and summarized in Equation (3):

|  | $\Delta G_{press}=k_{B}T\ln\left( \frac{p_{1}}{p_{0}} \right)=7.9 kJ/mol$ | (3) |
| --- | --- | --- |

where $k_{B}$ is the Boltzmann constant, $T$ is the absolute temperature ($T=300 K$), $p_{0}$ is the pressure for the ideal gas of 1 atm, and $p_{1}$ is the pressure of an ideal gas with a density of 1 mol/L ($p_{1}=24.6 atm$). This term is not a function of solvent composition; therefore, $7.9 kJ/mol$ is added to all simulated solvation free energies and does not influence the transfer free energies, which are computed as a difference between solvation free energies in the main text.

The third correction term deals with the energy required to transfer an ion from vacuum to bulk solution, passing through an interfacial potential [9], shown in Equation 4.

|  | $\Delta G_{surf}=ze\psi_{S}$ | (4) |
| --- | --- | --- |

where $z$ is the charge and $\psi_{S}$ is the interfacial potential between vacuum and bulk solvent. We computed $\psi_{S}$ in a separate MD simulation for different solvent systems (Section 2.2.3). $\Delta G_{surf}$ is a significant correction term with values ranging from 20 kJ/mol to 60 kJ/mol, depending on $\psi_{S}$. For instance, $\psi_{S}$ was computed to be -0.6 V for pure water, resulting in $\Delta G_{surf}=-58.7 kJ/mol$ for the hydronium ion. All free energies reported in this work include the three correction factors. Despite some significant corrections in the solvation free energies (up to ~90 kJ/mol), we note that $\Delta G_{sim}$ and $\Delta G_{solv}$ are linearly correlated suggesting that trends in the solvation free energies do not change upon inclusion of the correction factors (raw simulation data available in Supplementary Table 4).

### Box length

The box length is required for the calculation of $\Delta G_{fs}$. We computed the ensemble-average box length using the simulation window for which $\lambda_{vdW}= \lambda_{coul}=1.00$. The ensemble-average volume was computed from the 11 ns production run and the length was defined as the cube root of the volume.

### Dielectric constants

The dielectric constant is required for the calculation of $\Delta G_{fs}.$ We calculated the dielectric constant for pure and mixed solvent systems (in the absence of a solute) by using the solvent systems described in the Methods section of the main text and performing a 500 ps *NPT* equilibration and 50 ns *NPT* production simulation at *T =* 300 K and *P =* 1 bar. The last 40 ns of the production simulation trajectory was used to compute the dielectric constant using the GROMACS tool *gmx dipoles*. Supplementary Figure 2 compares the simulated dielectric constants to experimental data for pure solvents and aqueous mixtures of DIOX and DMSO. We find that the dielectric constant calculation is reasonably accurate for most pure solvent systems, with the largest error for GVL (80.1% error) and DMSO solvents (41.6% error). We note that optimizing force fields for MD simulations to obtain accurate dielectric constant remains a relevant challenge [10]. In aqueous mixtures of DIOX, the dielectric constant linearly decreases with increasing mass fraction of the organic phase in good agreement with experiments. In aqueous mixtures of DMSO, we do not capture the nonlinear decrease of the dielectric constant found in experiments, but we do capture similar overall values.


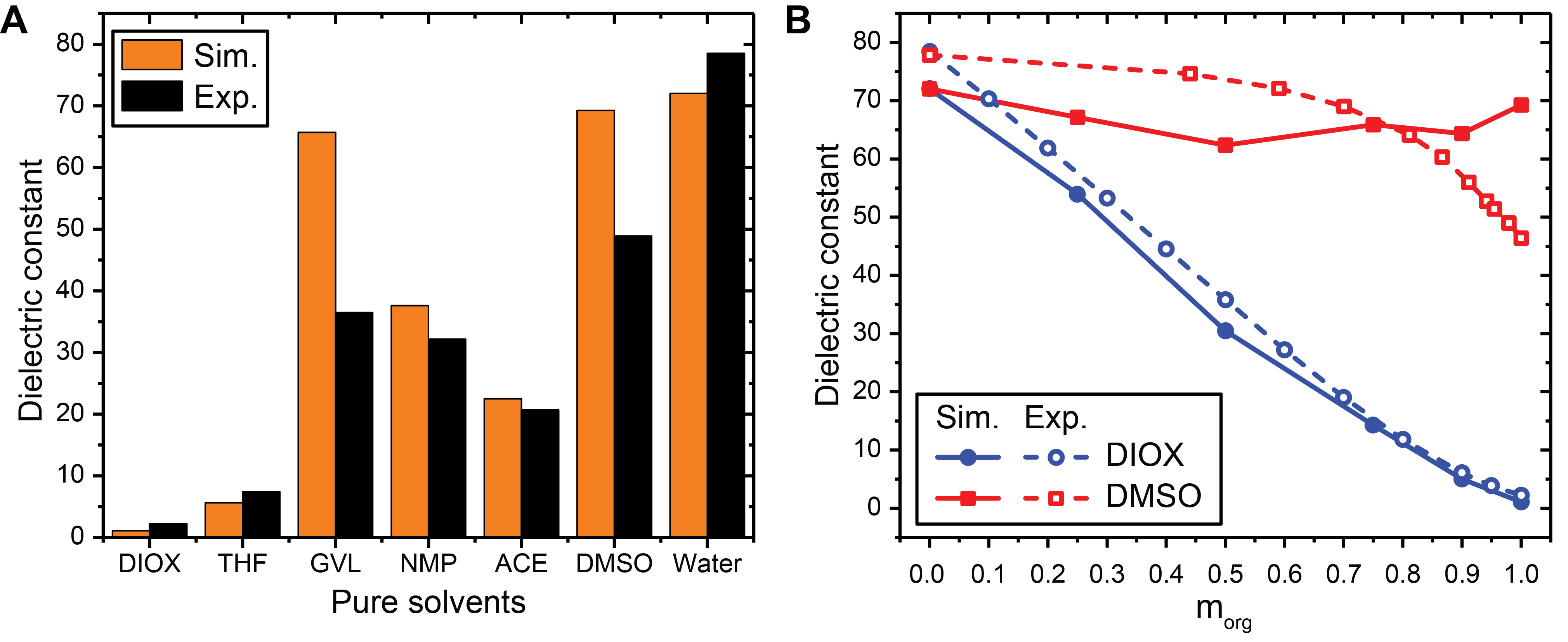


**Supplementary Figure 2.** **A** Simulation-derived (orange bars) and literature (black bars) dielectric constants. Literature values for DIOX, THF, ACE, DMSO, and water are from Ref. [11]. Literature values for GVL and NMP are from Ref. [12] and [13], respectively. **B** Simulation-derived (filled lines) and literature (dashed lines) dielectric constants for aqueous mixtures of DIOX and DMSO. Literature values for aqueous mixtures of DIOX and DMSO are from Ref. [14] and [15], respectively.

### Electrostatic potentials

The electrostatic potential difference at the vapor-liquid interface is required for the calculation of $\Delta G_{surf}$. The final simulation configurations from the production simulations used for the dielectric constant calculations were used to initialize additional simulations for electrostatic potential calculations. The simulation box was expanded by 8 nm in the z-direction to create a vapor region and a 20 ns *NVT* production simulation was then performed. The final 15 ns of the production data were used to compute electrostatic potentials using the GROMACS tool *gmx potential*, which numerically integrates the Poisson’s equation twice [9], shown in Equations 5 and 6:

|  | $E_{z}(z)=\int_{-\infty}^{z} \frac{\left\langle\rho(z^{'}) \right\rangle}{\epsilon_{0}}dz'$ | (5) |
| --- | --- | --- |
|  | $\psi(z)=\int_{-\infty}^{z} E_{z}(z)dz'$ | (6) |

where $\rho(z^{'})$ is the charge density, the brackets around $\rho(z^{'})$ indicate ensemble average, $\epsilon_{0}$ is the permittivity of vacuum ($\epsilon_{0}=8.85419\times{10}^{-12}\frac{C}{V\cdot m}$ ), $E_{z}(z)$ is the electric field in $\frac{V}{nm}$, and $\psi(z)$ is the electrostatic potential in $V$. Prior to computing the electrostatic potential, the solvent system was centered in the z‑direction and the solvent density as a function of z was computed using the GROMACS tool *gmx density* to estimate the location of the bulk solvent. Supplementary Figure 3 shows the electrostatic potential and solvent density as a function of z for pure water with a bin width of 0.02 nm. We normalize the density by its largest value and consider the upper 95% of the density as the “bulk solvent”. The interfacial potential is then defined as the difference in the electrostatic potential between vacuum (0) and the average electrostatic potential computed for the bulk solvent.


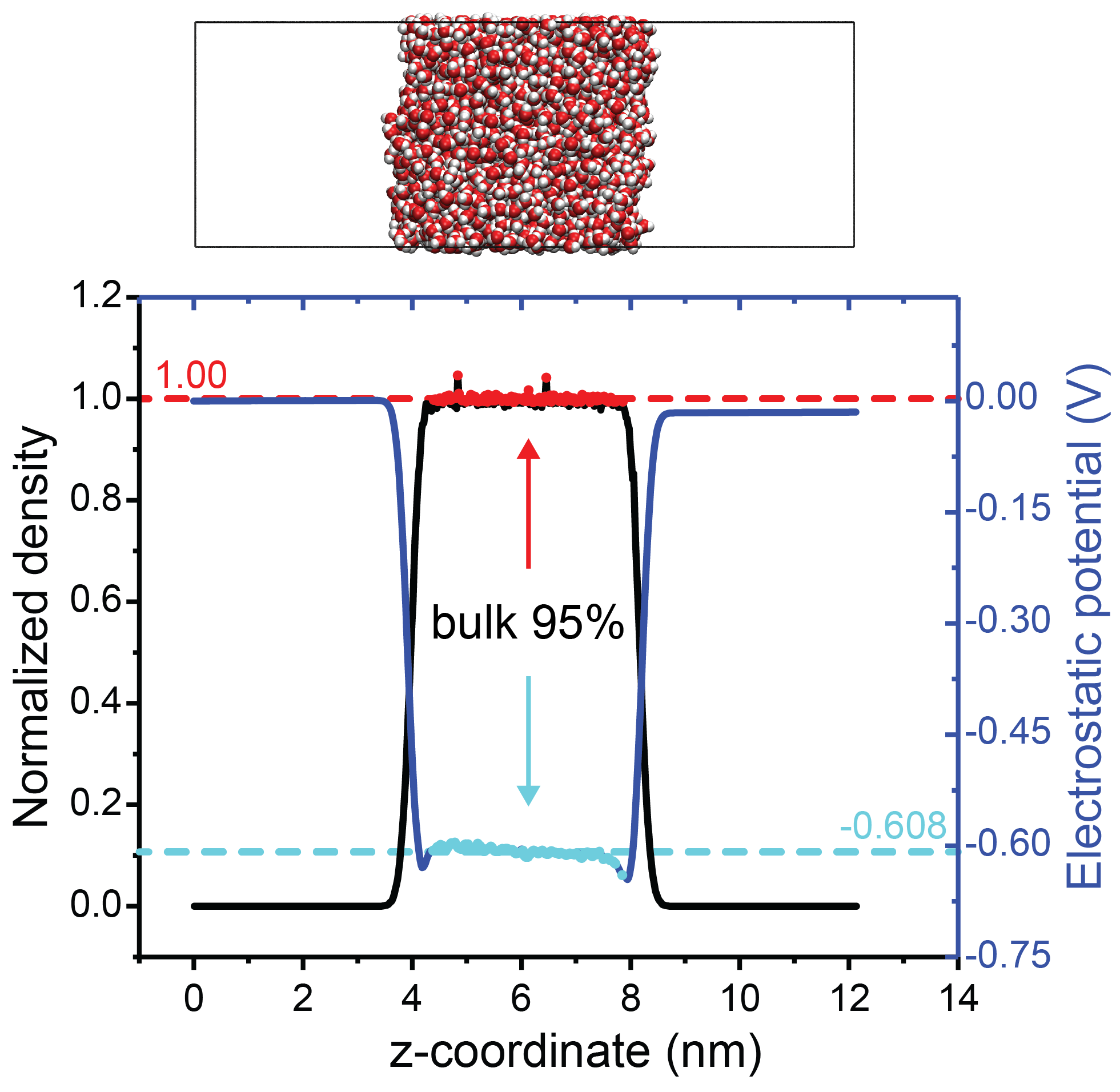


**Supplementary Figure 3.** Normalized density and electrostatic potential as a function of z-coordinate for pure water. Dotted red and cyan points indicate the upper 95% of the density. The bin width used was 0.02 nm. Dashed red and cyan lines indicate the average bulk density and electrostatic potential, respectively. A simulation snapshot of water with periodic boundaries drawn as black lines is shown above the plot.

## Tabulated system size, dielectric constant, and electrostatic potentials

Supplementary Table 2 summarizes the box length, dielectric constant, and electrostatic potential used for the correction factors in the free energy calculations for pure solvents. Supplementary Table 3 summarizes the same parameters for aqueous mixtures of DIOX and DMSO.

**Supplementary Table 2**. Simulation system information for the pure solvent systems. $N$ is the total number of solvent molecules in the system. $L_{H_{3}O^{+}} (\mathrm{nm})$ and $L_{Cl^{-}} (\mathrm{nm})$ are ensemble-average box lengths for the hydronium and chlorine ions, respectively. Dielectric constants and electrostatic potentials were computed as discussed in Sections 2.2.2 and 2.2.3. Box lengths and dielectric constants were used for computing the correction term for finite-size effects in Equation 2. Electrostatic potentials were used to compute the correction term for interfacial potentials in Equation 4.

| Solvent | $N$ | $L_{H_{3}O^{+}} (nm)$ | $L_{Cl^{-}} (nm)$ | Dielectric constant | Electrostatic potential (V) |
| --- | --- | --- | --- | --- | --- |
| Water | 2400 | 4.16 | 4.17 | 72.02 | -0.608 |
| DIOX | 900 | 5.03 | 5.04 | 1.09 | -0.415 |
| THF | 1350 | 5.70 | 5.69 | 5.62 | -0.567 |
| GVL | 720 | 4.90 | 4.90 | 65.70 | -0.346 |
| NMP | 675 | 4.81 | 4.81 | 37.59 | -0.534 |
| ACE | 1350 | 5.51 | 5.51 | 22.50 | -0.272 |
| DMSO | 981 | 4.88 | 4.88 | 69.27 | -0.498 |

**Supplementary Table 3.** Simulation system information for aqueous mixtures of DIOX and DMSO. $m_{org}$ is the mass fraction of the organic phase. $N_{org}$ and $N_{H_{2}O}$ are the number of organic cosolvent and water molecules. $L_{H_{3}O^{+}} (\mathrm{nm})$ and $L_{Cl^{-}} (\mathrm{nm})$ are ensemble-average box lengths for the hydronium and chlorine ions, respectively. Dielectric constants and electrostatic potentials were computed as discussed in Sections 2.2.2 and 2.2.3. Box lengths and dielectric constants were used for computing the correction term for finite-size effects in Equation 2. Electrostatic potentials were used to compute the correction term for interfacial potentials in Equation 4.

| Cosolvent | $m_{org}$ | $N_{org}$ | $N_{H_{2}O}$ | $L_{H_{3}O^{+}}$  $(nm)$ | $L_{Cl^{-}}$  $(nm)$ | Dielectric constant | Electrostatic potential (V) |
| --- | --- | --- | --- | --- | --- | --- | --- |
| DIOX | 0.90 | 748 | 403 | 4.91 | 4.90 | 5.00 | -0.517 |
|  | 0.75 | 558 | 911 | 4.72 | 4.72 | 14.27 | -0.673 |
|  | 0.50 | 317 | 1552 | 4.50 | 4.50 | 30.46 | -0.779 |
|  | 0.25 | 130 | 2050 | 4.31 | 4.29 | 53.97 | -0.820 |
| DMSO | 0.90 | 816 | 402 | 4.75 | 4.76 | 64.38 | -0.589 |
|  | 0.75 | 617 | 889 | 4.61 | 4.61 | 65.86 | -0.687 |
|  | 0.50 | 357 | 1525 | 4.42 | 4.42 | 62.36 | -0.682 |
|  | 0.25 | 152 | 2027 | 4.28 | 4.28 | 67.15 | -0.681 |

## Sample calculation of free energy correction terms

We show a sample calculation for the three correction terms for transferring a hydronium ion from vacuum to pure water. The finite-sized correction factor is computed using Equation 2, using dielectric constant and box length from Supplementary Table 2:

$$\Delta G_{fs}=\frac{e^{2}\left( 72.02-1 \right)}{6\left( 72.02 \right)\left( 5.727\times{10}^{-4}\frac{e^{2}\mathrm{mol}}{\mathrm{kJ}\cdot\mathrm{nm}} \right)\left( 4.16 \mathrm{nm} \right)}\left[ \left( \frac{0.100 \mathrm{nm}}{4.16 \mathrm{nm}} \right)^{2}-\frac{4\pi}{15}\left( \frac{0.100 \mathrm{nm}}{4.16 \mathrm{nm}} \right)^{5} \right] -\frac{e^{2}\left( -2.837297 \right)}{8\pi\left( 72.02 \right)\left( 5.727\times{10}^{-4}\frac{e^{2}\mathrm{mol}}{\mathrm{kJ}\cdot\mathrm{nm}} \right)\left( 4.16 \mathrm{nm} \right)}= 0.6978\frac{\mathrm{kJ}}{\mathrm{mol}}$$

The pressure correction is the same for all compositions ($\Delta G_{press}=7.9 kJ/mol$). The free energy correction to correct for passing through a vapor-liquid interface is computed using Equation 4, using the electrostatic potential from Supplementary Table 2:

$$\Delta G_{surf}=\left( +1 \right)\left( 1.602176487\times{10}^{-19}C \right)\left( -0.608 V \right)\left( \frac{1 J}{1 C\cdot V} \right)\left( \frac{1 \mathrm{kJ}}{{10}^{3}J} \right)\left( \frac{6.022\times{10}^{23}}{1 \mathrm{mol}} \right)=-58.6\frac{\mathrm{kJ}}{\mathrm{mol}}$$

## Correction terms for the hydronium ion

Supplementary Figure 4 shows each of the three correction factors for the hydronium ion solvation free energy in each pure solvent. We find that the finite-size correction ($\Delta G_{fs}$) is low for most solvents, except for DIOX and THF, which are the solvents with the lowest dielectric constant (Supplementary Figure 2A). The most significant correction term is the interfacial potential ($\Delta G_{surf}$) which ranges from -25 to -59 kJ/mol. Supplementary Table 4 lists each of these contributions and the solvation free energy.


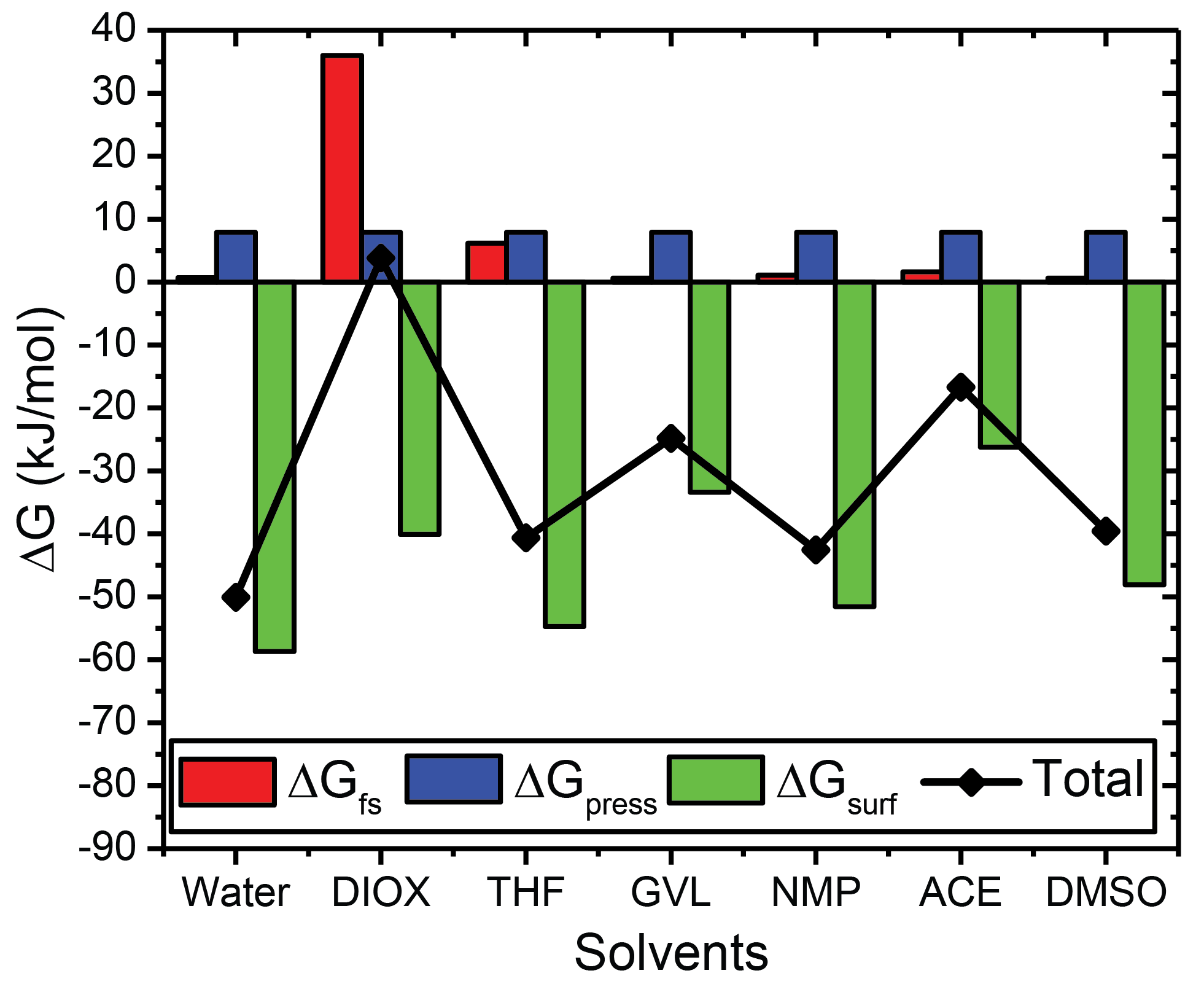


**Supplementary Figure 4.** Correction factors for finite-size effects ($\Delta G_{fs}$), pressure ($\Delta G_{press}$), and interfacial potential ($\Delta G_{surf})$ for the solvation free energy of hydronium ion to pure solvents. The black line indicates the sum of all correction factors.

**Supplementary Table 4.** Simulated solvation free energy results for the hydronium and chloride ions in pure solvent systems. $\Delta G_{sim} (\mathrm{avg})$ and $\Delta G_{sim} (\mathrm{err})$ are free energies calculated from the average and standard deviation of two trials. The simulated free energy is computed with the Multistate Bennett Acceptance Ratio method [16], available in the python alchemical analysis tool [17]. $\Delta G_{fs}$, $\Delta G_{press}$, $\Delta G_{surf}$ are correction terms defined in Equation 1. $\Delta G_{solv}$ is the solvation free energy as reported in the main text. All $\Delta G$ values are in units of $kJ/mol$.

| Hydronium ion | | | | | | |
| --- | --- | --- | --- | --- | --- | --- |
| Solvent | $\Delta G_{sim} (\mathrm{avg})$ | $\Delta G_{sim} (\mathrm{err})$ | $\Delta G_{fs}$ | $\Delta G_{press}$ | $\Delta G_{surf}$ | $\Delta G_{solv}$ |
| Water | -414.99 | 0.43 | 0.70 | 7.90 | -58.68 | -465.07 |
| DIOX | -284.31 | 0.07 | 35.98 | 7.90 | -40.06 | -280.49 |
| THF | -371.47 | 0.46 | 6.17 | 7.90 | -54.71 | -412.11 |
| GVL | -402.48 | 0.20 | 0.64 | 7.90 | -33.37 | -427.31 |
| NMP | -426.86 | 0.69 | 1.12 | 7.90 | -51.56 | -469.41 |
| ACE | -459.53 | 0.16 | 1.61 | 7.90 | -26.21 | -476.23 |
| DMSO | -488.97 | 0.43 | 0.61 | 7.90 | -48.08 | -528.54 |
|  |  |  |  |  |  |  |
| Chloride ion | | | | | | |
| Solvent | $\Delta G_{sim} (avg)$ | $\Delta G_{sim} (err)$ | $\Delta G_{fs}$ | $\Delta G_{press}$ | $\Delta G_{surf}$ | $\Delta G_{solv}$ |
| Water | -353.79 | 0.05 | 0.79 | 7.90 | 58.68 | -286.43 |
| DIOX | -165.00 | 0.14 | 35.88 | 7.90 | 40.06 | -81.16 |
| THF | -193.29 | 0.20 | 6.21 | 7.90 | 54.71 | -124.48 |
| GVL | -231.78 | 0.50 | 0.69 | 7.90 | 33.37 | -189.82 |
| NMP | -241.47 | 0.04 | 1.17 | 7.90 | 51.56 | -180.84 |
| ACE | -211.46 | 0.15 | 1.64 | 7.90 | 26.21 | -175.71 |
| DMSO | -237.91 | 0.17 | 0.66 | 7.90 | 48.08 | -181.26 |

Supplementary Figure 5 shows each of the three correction factors for the hydronium ion solvation free energy in aqueous mixtures of DIOX and DMSO. Across solvent compositions, the dominating correction term is the interfacial potential ($\Delta G_{surf}$). The total correction is non-monotonic as a function of organic mass fraction. Supplementary Table 5 lists each of these contributions and the solvation free energy.


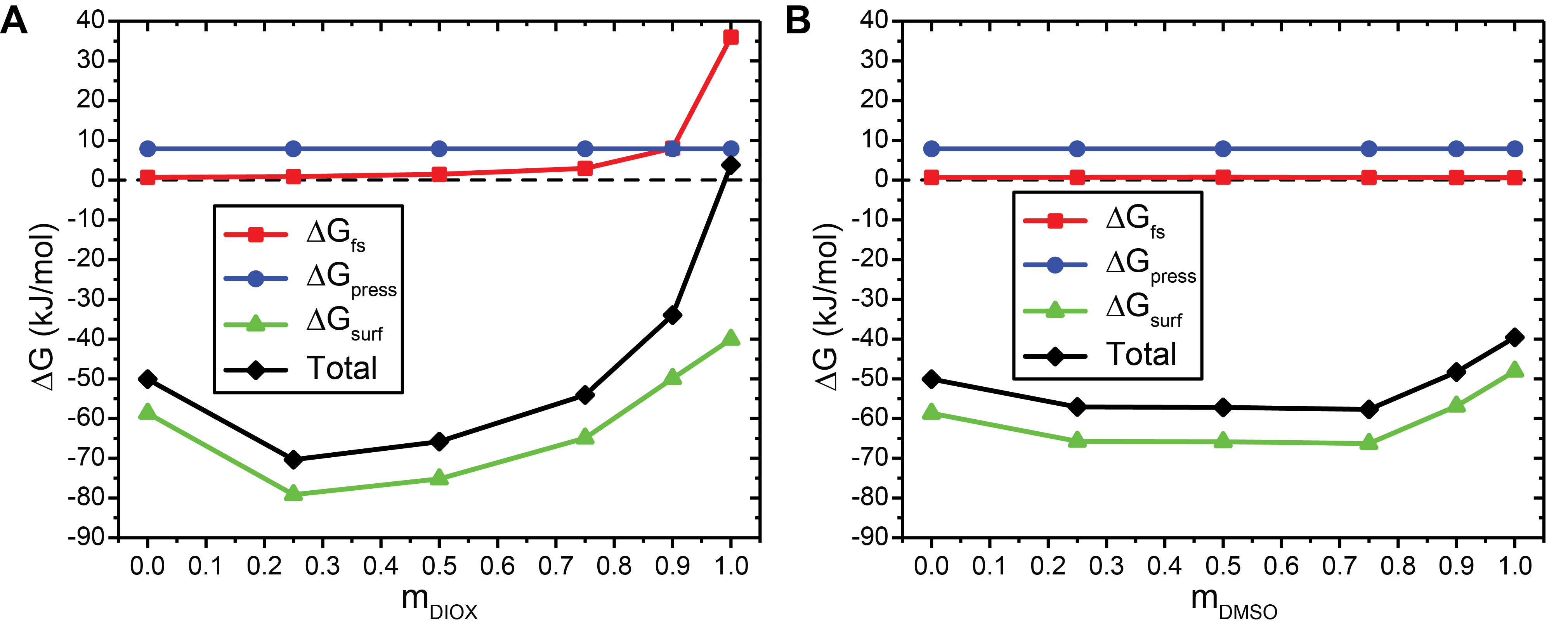


**Supplementary Figure 5.** Correction factors for finite-size effects ($\Delta G_{fs}$), pressure ($\Delta G_{press}$), and interfacial potential ($\Delta G_{surf})$ for the hydronium ion solvation free energy in aqueous mixtures of **A** DIOX and **B** DMSO as a function of organic mass fraction. The black line indicates the sum of all correction factors.

**Supplementary Table 5.** Solvation free energy contributions for the hydronium and chloride ions in aqueous mixtures of DIOX and DMSO. $\Delta G_{sim} (\mathrm{avg})$ and $\Delta G_{sim} (\mathrm{err})$ are free energies calculated from the average and standard deviation of two trials. The simulated free energy is computed with the Multistate Bennett Acceptance Ratio method [16], available in the python alchemical analysis tool [17]. $\Delta G_{fs}$, $\Delta G_{press}$, $\Delta G_{surf}$ are correction terms defined in Equation 1. $\Delta G_{solv}$ is the solvation free energy as reported in the main text. All $\Delta G$ values are in units of $kJ/mol$.

| Hydronium ion | | | | | | | |
| --- | --- | --- | --- | --- | --- | --- | --- |
| Cosolvent | $m_{org}$ | $\Delta G_{sim} (avg)$ | $\Delta G_{sim} (err)$ | $\Delta G_{fs}$ | $\Delta G_{press}$ | $\Delta G_{surf}$ | $\Delta G_{solv}$ |
| DIOX | 0.90 | -445.86 | 2.06 | 8.05 | 7.90 | -49.91 | -479.82 |
|  | 0.75 | -443.67 | 0.19 | 2.95 | 7.90 | -64.91 | -497.72 |
|  | 0.50 | -431.85 | 0.92 | 1.47 | 7.90 | -75.17 | -497.65 |
|  | 0.25 | -421.53 | 0.12 | 0.88 | 7.90 | -79.13 | -491.88 |
| DMSO | 0.90 | -477.51 | 0.74 | 0.67 | 7.90 | -56.85 | -525.79 |
|  | 0.75 | -458.45 | 0.54 | 0.68 | 7.90 | -66.28 | -516.15 |
|  | 0.50 | -443.77 | 0.09 | 0.75 | 7.90 | -65.83 | -500.96 |
|  | 0.25 | -426.79 | 0.44 | 0.72 | 7.90 | -65.71 | -483.88 |
|  |  |  |  |  |  |  |  |
| Chlorine ion | | | | | | | |
| Cosolvent | $m_{org}$ | $\Delta G_{sim} (avg)$ | $\Delta G_{sim} (err)$ | $\Delta G_{fs}$ | $\Delta G_{press}$ | $\Delta G_{surf}$ | $\Delta G_{solv}$ |
| DIOX | 0.90 | -277.86 | 2.53 | 8.12 | 7.90 | 49.91 | -211.93 |
|  | 0.75 | -301.15 | 0.06 | 3.01 | 7.90 | 64.91 | -225.34 |
|  | 0.50 | -325.60 | 0.92 | 1.54 | 7.90 | 75.17 | -240.99 |
|  | 0.25 | -342.51 | 0.09 | 0.97 | 7.90 | 79.13 | -254.51 |
| DMSO | 0.90 | -256.28 | 0.23 | 0.73 | 7.90 | 56.85 | -190.80 |
|  | 0.75 | -282.44 | 2.38 | 0.75 | 7.90 | 66.28 | -207.51 |
|  | 0.50 | -316.27 | 0.05 | 0.82 | 7.90 | 65.83 | -241.71 |
|  | 0.25 | -336.39 | 0.03 | 0.81 | 7.90 | 65.71 | -261.97 |

# 1,2-propanediol dehydration reaction

## Experimental reaction kinetics for 1,2-propanediol dehydration

Experimental rate constants for 1,2-propanediol dehydration were taken from Ref. [18] and are listed in Supplementary Table 6. We summarize the data for aqueous mixtures of DIOX and DMSO. We compute $\sigma$ using Equation 2 of the main text.

**Supplementary Table 6.** Experimental reaction kinetics for 1,2-propanediol acid-catalyzed dehydration reaction taken from Ref. [18]. $k_{org}/k_{H_{2}O}$ is the ratio of rate constants between aqueous mixtures of the organic phase and pure water. $\sigma$ is the kinetic solvent parameter, as defined in Equation 3 of the main text.

| DIOX-water mixtures | | | | |
| --- | --- | --- | --- | --- |
| $m_{org}$ |  | $k_{org}/k_{H_{2}O}$ |  | $\sigma$ |
| 0.00 |  | 1.00 |  | 0.00 |
| 0.25 |  | 0.94 |  | -0.03 |
| 0.50 |  | 0.67 |  | -0.17 |
| 0.75 |  | 1.22 |  | 0.09 |
| 0.90 |  | 3.73 |  | 0.57 |
| 0.97 |  | 19.83 |  | 1.30 |
| 0.99 |  | 22.68 |  | 1.36 |
|  | | | | |
| DMSO-water mixtures | | | | |
| $m_{org}$ |  | $k_{org}/k_{H_{2}O}$ |  | $\sigma$ |
| 0.00 |  | 1.00 |  | 0.00 |
| 0.25 |  | 0.49 |  | -0.31 |
| 0.50 |  | 1.05 |  | 0.02 |
| 0.75 |  | 3.60 |  | 0.56 |
| 0.90 |  | 11.15 |  | 1.05 |
| 0.98 |  | 42.62 |  | 1.63 |

## Hypothesized reaction mechanism of 1,2-propanediol to propanal

The hypothesized reaction mechanism for 1,2-propanediol dehydration to propanal is reproduced below from Ref. [18]:

|  | $CH_{2}\left( \mathrm{OH} \right)\mathrm{CH}\left( \mathrm{OH} \right)CH_{3(aq)}+H_{(aq)}^{+}\to CH_{2}\mathrm{OHCH}\left( OH_{2}^{+} \right)CH_{3(aq)}$ | (7) |
| --- | --- | --- |
|  | $CH_{2}\mathrm{OHCH}\left( OH_{2}^{+} \right)CH_{3(aq)}\to CH_{2}\mathrm{OHCHC}H_{3\left( \mathrm{aq} \right)}^{+}+H_{2}O_{(aq)}$ | (8) |
|  | $CH_{2}\mathrm{OHCHC}H_{3\left( \mathrm{aq} \right)}^{+}+H_{2}O_{(aq)}\underset{\to}{\text{H-shift}}\mathrm{CHOC}H_{2}CH_{3\left( \mathrm{aq} \right)}+H_{3}O_{(aq)}^{+}$ | (9) |

# Additional simulation results

## Radial distribution function of cosolvents for 1,2-propanediol

Supplementary Figure 6 shows the radial distribution function (RDF) between 1,2-propanediol and organic cosolvents for 90 wt% DMSO and 90 wt% DIOX. We find only minor differences between the RDFs of the two cosolvents.


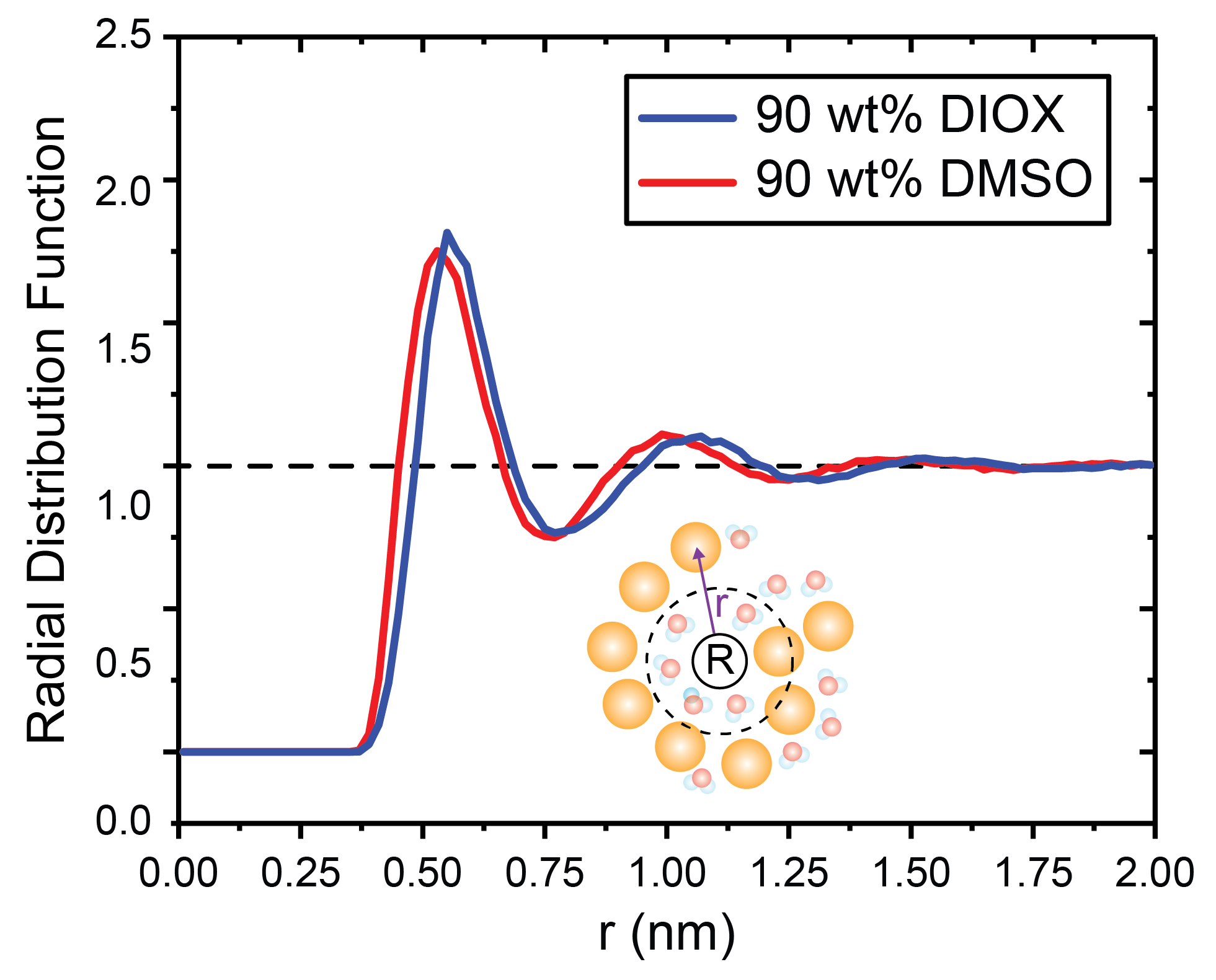


**Supplementary Figure 6.** Radial distribution function between 1,2-propanediol and organic solvents in 90 wt% DIOX and 90 wt% DMSO mixtures.

## Radial distribution function of hydronium ion in pure solvents

Supplementary Figure 7 shows the RDF between the hydronium ion and water in each pure solvent. The location of the first solvation shell appears to be dictated by molecular size, where water has the smallest peak distance and GVL has the largest peak distance. ACE has the highest RDF peak height, indicating a large likelihood of finding ACE at the first solvation shell. The RDFs for DIOX and DMSO vary slightly in terms of peak height and distance. Overall, the RDF alone cannot explain trends found in Figure 3C of the main text.


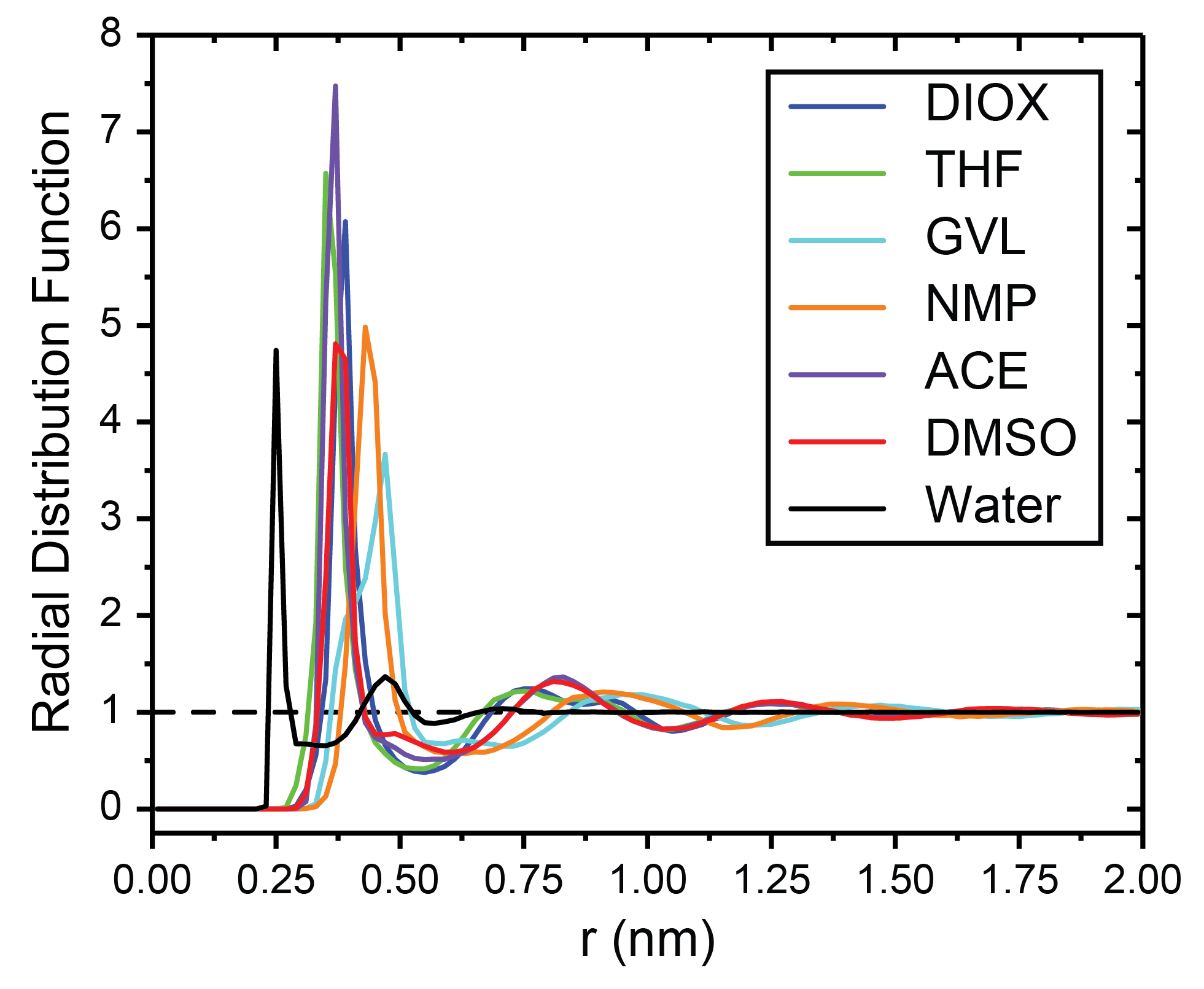


**Supplementary Figure 7.** Radial distribution function between the center of mass of the hydronium ion and solvent molecules in pure water and organic solvent systems.

# Correlating simulation-derived observables to experimental reaction rates

Supplementary Figure 8 shows the correlation between predicted kinetic solvent parameter ($\sigma_{pred}$) and experimental kinetic solvent parameter ($\sigma_{exp}$) using Equation 7 of the main text. The best-fit slope is 0.25 and the root-mean-square error (RMSE) between predicted and experimental values is 0.39. We find poor correlations in DMSO-water mixtures, which we attribute to missing information in the correlative model, motivating the development of Equation 9 in the main text.


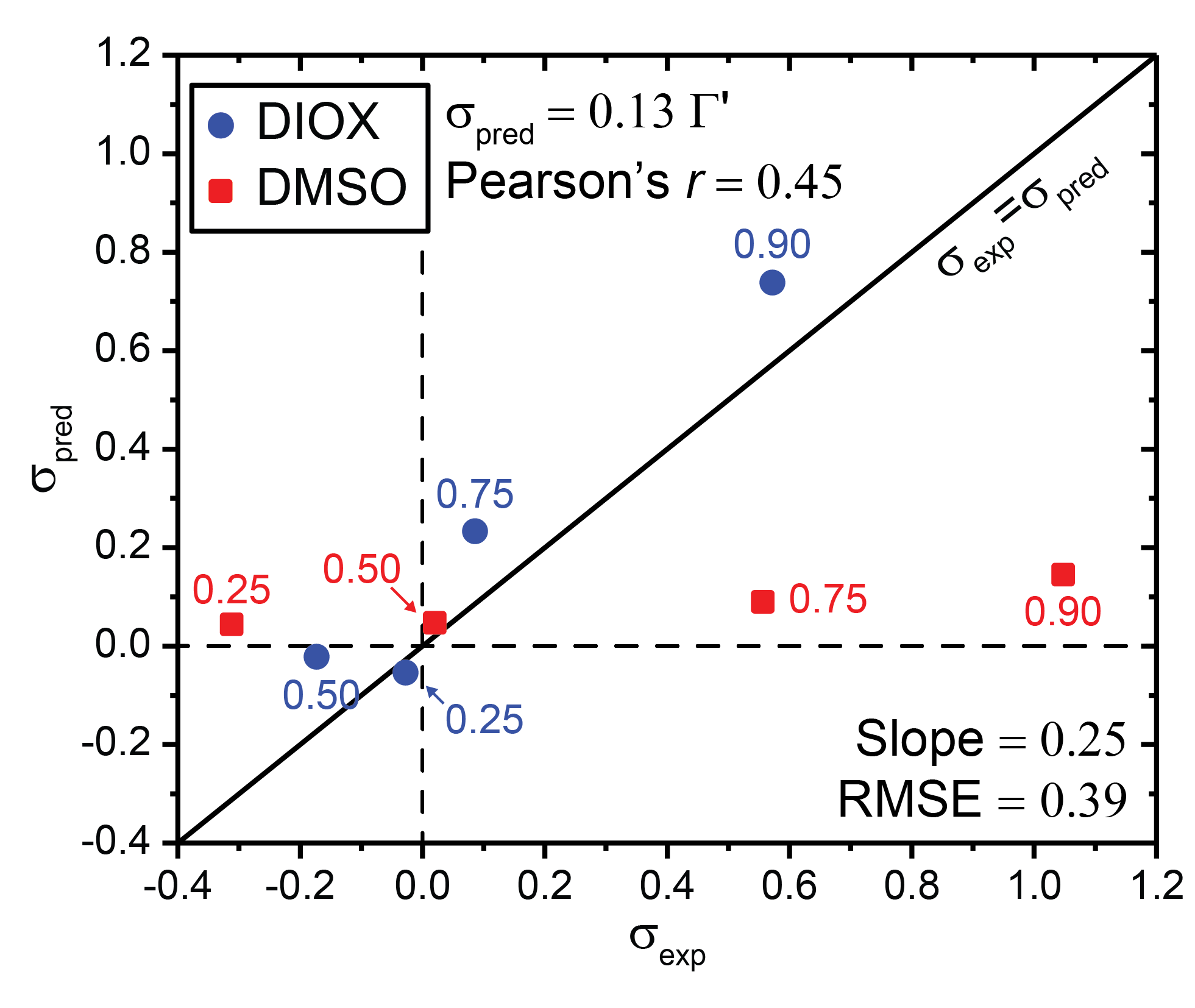


**Supplementary Figure 8.** Parity plot between predicted kinetic solvent parameter ($\sigma_{pred}$) and experimental kinetic solvent parameter ($\sigma_{exp}$) using results from aqueous mixtures of DIOX and DMSO. The correlative model is based on Equation 7 as shown within the plot. Data points are labeled with the wt% of the organic solvent.

## Ratio of hydronium ion transfer free energy

Supplementary Figure 9 shows the ratio of hydronium ion solvation free energies ($\Delta G_{H_{3}O^{+}}^{k/H_{2}O}$), as defined in Equation 8 of the main text.


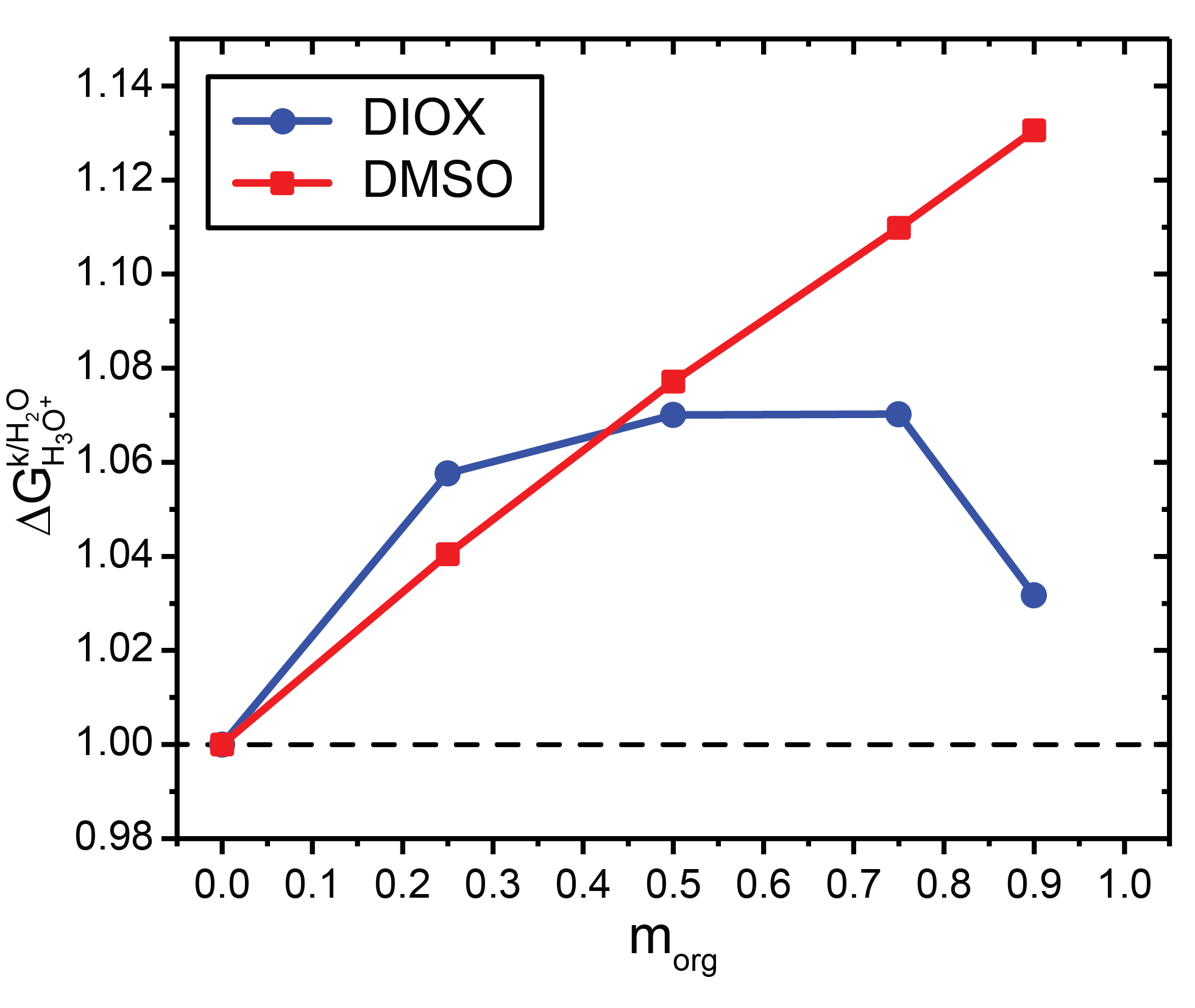


**Supplementary Figure 9.** Ratio of the hydronium ion transfer free energy in the $k$th solvent system ($\Delta G_{H_{3}O^{+}}^{k}$) to pure water ($\Delta G_{H_{3}O^{+}}^{H_{2}O}$) for various mass fractions of DIOX-water and DMSO-water mixtures.

## Details on multilinear regression model

Variables were normalized so weights are comparable in multilinear regression equations shown in Equation 10.

|  | $\hat{x}=\frac{x-\mu}{\sigma}$ | (10) |
| --- | --- | --- |

where $x$ is the variable, $\mu$ is the mean, $\sigma$ is the standard deviation, and $\hat{x}$ is the normalized variable. Supplementary Table 7 shows the data used for the multilinear regression model in Equation 9 of the main text. We selected the model based on minimizing the Akaike Information Criterion with a correction term for small sample sizes (AIC_c_), described in our previous work [19].

**Supplementary Table 7.** Data used for the multilinear regression model in Equation 9 of the main text. $\Gamma$ is the preferential exclusion coefficient described in Equation 2 of the main text. $\Gamma^{'}$ includes a correction term described in Equation 7 of the main text. $\Delta G_{H_{3}O^{+}}^{k/H_{2}O}$is the ratio of solvation free energy of the hydronium in $k$th solvent system to the solvation free energy of hydronium ion in pure water, described in Equation 8 of the main text. $\hat{\Gamma^{'}}$ and $\hat{\Delta G_{H_{3}O^{+}}^{k/H_{2}O}}$ are normalized variables based on Equation 10. $\sigma_{exp}$ is the experimental kinetic solvent parameter described in Equation 3 of the main text. $\sigma_{pred}$ is the predicted kinetic solvent parameter based on the model described in Equation 9 of the main text.

|  |  |  |  |  |  | Normalized variables | |  |  |  |
| --- | --- | --- | --- | --- | --- | --- | --- | --- | --- | --- |
| Cosolvent | m_org_ | $\Gamma$ | $\Gamma^{'}$ | $\Delta G_{H_{3}O^{+}}^{k/H_{2}O}$ |  | $\hat{\Gamma^{'}}$ | $\hat{\Delta G_{H_{3}O^{+}}^{k/H_{2}O}}$ |  | $\sigma_{exp}$ | $\sigma_{pred}$ |
| DIOX | 0.90 | 5.52 | 5.52 | 1.03 |  | 2.47 | -1.35 |  | 0.57 | 0.51 |
|  | 0.75 | 1.75 | 1.75 | 1.07 |  | 0.34 | -0.10 |  | 0.09 | 0.29 |
|  | 0.50 | -0.16 | -0.16 | 1.07 |  | -0.74 | -0.11 |  | -0.17 | -0.06 |
|  | 0.25 | -0.40 | -0.40 | 1.06 |  | -0.87 | -0.51 |  | -0.03 | -0.26 |
| DMSO | 0.90 | -1.09 | 1.09 | 1.13 |  | -0.03 | 1.84 |  | 1.05 | 0.92 |
|  | 0.75 | -0.67 | 0.67 | 1.11 |  | -0.27 | 1.17 |  | 0.56 | 0.59 |
|  | 0.50 | -0.36 | 0.36 | 1.08 |  | -0.45 | 0.12 |  | 0.02 | 0.12 |
|  | 0.25 | -0.33 | 0.33 | 1.04 |  | -0.46 | -1.06 |  | -0.31 | -0.34 |

# Temperature dependence of hydronium solvation free energies

Supplementary Figure 10 shows the hydronium ion solvation free energy in pure water, DIOX, and DMSO without accounting for any correction terms from Section 2.2. These results show that hydronium ion is more stable in pure DMSO than DIOX relative to pure water, regardless of the temperature between 300 K and 433.15 K (the reaction temperature for 1,2-propanediol dehydration). We expect that the inclusion of correction terms would not significantly change these trends.


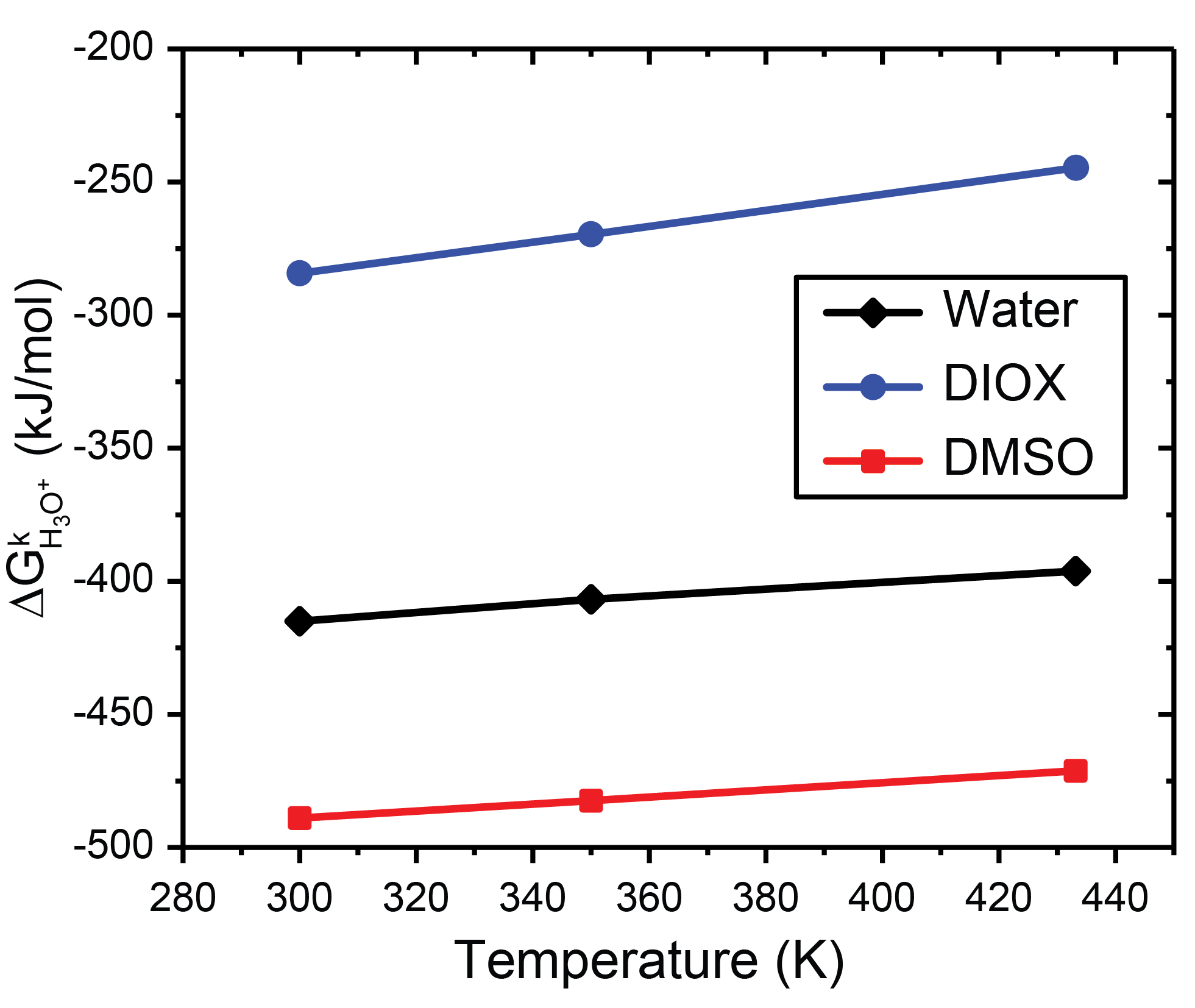


**Supplementary Figure 10.** Solvation free energies of a hydronium ion in pure water, DIOX, and DMSO systems at 300 K, 350 K, and 433.15 K. Solvation free energies for 350 K and 433.15 K were computed with shorter 5 ns *NPT* simulations compared to the 11 ns *NPT* simulations in the main text. These solvation free energies do not take into account any correction terms as described in Section 2.2.

# References

1. Bonthuis, D.J., S.I. Mamatkulov, and R.R. Netz, *Optimization of classical nonpolarizable force fields for OH- and H3O+.* Journal of Chemical Physics, 2016. **144**.

2. Hess, B., et al., *LINCS: A linear constraint solver for molecular simulations.* Journal of Computational Chemistry, 1997. **18**(12): p. 1463-1472.

3. Beutler, T.C., et al., *Avoiding Singularities and Numerical Instabilities in Free-Energy Calculations Based on Molecular Simulations.* Chemical Physics Letters, 1994. **222**(6): p. 529-539.

4. Shivakumar, D., et al., *Prediction of Absolute Solvation Free Energies using Molecular Dynamics Free Energy Perturbation and the OPLS Force Field.* Journal of Chemical Theory and Computation, 2010. **6**(5): p. 1509-1519.

5. Rocklin, G.J., et al., *Calculating the binding free energies of charged species based on explicit-solvent simulations employing lattice-sum methods: An accurate correction scheme for electrostatic finite-size effects.* Journal of Chemical Physics, 2013. **139**(18).

6. Marcus, Y., *Volumes of aqueous hydrogen and hydroxide ions at 0 to 200 degrees C.* Journal of Chemical Physics, 2012. **137**(15).

7. Feakins, D. and P. Watson, *Studies in Ion Solvation in Non-Aqueous Solvents and Their Aqueous Mixtures .2. Properties of Ion Constituents.* Journal of the Chemical Society, 1963(Oct): p. 4734-&.

8. Bonthuis, D.J., S. Gekle, and R.R. Netz, *Dielectric Profile of Interfacial Water and its Effect on Double-Layer Capacitance.* Physical Review Letters, 2011. **107**(16).

9. Sedlmeier, F., et al., *Water at polar and nonpolar solid walls.* Biointerphases, 2008. **3**(3): p. Fc23-Fc39.

10. Liu, Z., et al., *Benchmarks and Dielectric Constants for Reparametrized OPLS and Polarizable Force Field Models of Chlorinated Hydrocarbons.* Journal of Physical Chemistry B, 2018. **122**(2): p. 770-779.

11. Fowler, F.W., A.R. Katritzky, and R.J. Rutherford, *Correlation of Solvent Effects on Physical and Chemical Properties.* Journal of the Chemical Society B-Physical Organic, 1971(3): p. 460-+.

12. Wohlfarth, C., *Static dielectric constant of γ-valerolactone*, in *Static Dielectric Constants of Pure Liquids and Binary Liquid Mixtures: Supplement to Volume IV/17*, M.D. Lechner, Editor. 2015, Springer Berlin Heidelberg: Berlin, Heidelberg. p. 91-91.

13. Uosaki, Y., K. Kawamura, and T. Moriyoshi, *Static relative permittivities of water plus 1-methyl-2-pyrrolidinone and water plus 1,3-dimethyl-2-imidazolidinone mixtures under pressures up to 300 MPa at 298.15 K.* Journal of Chemical and Engineering Data, 1996. **41**(6): p. 1525-1528.

14. Critchfield, F.E., J.A. Gibson, and J.L. Hall, *Dielectric Constant for the Dioxane Water System from 20 to 35-Degrees.* Journal of the American Chemical Society, 1953. **75**(8): p. 1991-1992.

15. Plowas, I., J. Swiergiel, and J. Jadzyn, *Relative Static Permittivity of Dimethyl Sulfoxide plus Water Mixtures.* Journal of Chemical and Engineering Data, 2013. **58**(6): p. 1741-1746.

16. Shirts, M.R. and J.D. Chodera, *Statistically optimal analysis of samples from multiple equilibrium states.* Journal of Chemical Physics, 2008. **129**(12).

17. Klimovich, P.V., M.R. Shirts, and D.L. Mobley, *Guidelines for the analysis of free energy calculations.* Journal of Computer-Aided Molecular Design, 2015. **29**(5): p. 397-411.

18. Mellmer, M.A., et al., *Solvent-enabled control of reactivity for liquid-phase reactions of biomass-derived compounds.* Nature Catalysis, 2018. **1**(3): p. 199-207.

19. Walker, T.W., et al., *Universal kinetic solvent effects in acid-catalyzed reactions of biomass-derived oxygenates.* Energy & Environmental Science, 2018. **11**(3): p. 617-628.
